# Supplementary material for: Uncovering hidden brain state dynamics that regulate performance and decision-making during cognition
Source: Nat Commun. 2018 Jun 27;9:2505. doi: 10.1038/s41467-018-04723-6 (PMC6021386; doi:10.1038/s41467-018-04723-6)
Supplement: Supplementary file 1 — Supplementary Information [file 41467_2018_4723_MOESM1_ESM.pdf]

Supplementary Information

Uncovering hidden brain state dynamics that regulate performance and decision-making during cognition

Jalil Taghia, Weidong Cai, Srikanth Ryali, John Kochalka,
Jonathan Nicholas, Tianwen Chen, Vinod Menon

Supplementary Methods

1 Bayesian switching dynamical systems

1.1 Model

Let $\boldsymbol{y}_t^s = (y_{t1}^s, \ldots, y_{1D}^s)^\top$ denote a D-dimensional vector of ROI timeseries measured from subject s in time t, where D is the number of ROIs and $\top$ denotes the transpose operator. We collect a sequence of T measurements from S subjects in $\underline{\boldsymbol{Y}} = \{\boldsymbol{Y}^s \mid s = 1, \ldots, S\}$ where $\boldsymbol{Y}^s = \{\boldsymbol{y}_1^s, \ldots, \boldsymbol{y}_T^s\}$.

Following the general formulation of the switching state-space models, we define $\boldsymbol{z}_t^s$ as the *latent state variables* and $\boldsymbol{x}_{kt}^s$ as the *latent space variables* associated to $\boldsymbol{y}_t^s$ at the k-th latent state, that is $z_{kt}^s = 1$. The set of latent state and latent space variables are shown by $\underline{\boldsymbol{Z}} = \{\boldsymbol{Z}^s \mid s = 1, \ldots, S\}$ and $\underline{\boldsymbol{X}} = \{\boldsymbol{X}_k^s \mid s = 1, \ldots, S,\ k = 1, \ldots, K\}$, respectively. The latent state variable $\boldsymbol{z}_t^s$ is a 1–of–K discrete vector with elements of $z_{kt}^s,\ \forall k = 1, \ldots, K$. As shown in the left panel of **Supplementary Figure** 1, two consecutive time instances are dependent via a first-order Markov chain through a hidden Markov model (HMM). HMMs are statistical Markov models which have been extensively used in various applications for modeling timeseries[1,2,3] including dynamic functional connectivity analysis of the fMRI data[4,5,6,7]. Let elements of the HMM model be defined as:

- initial state probability distribution, $\boldsymbol{\pi} = \left\{\pi_k \mid \sum_{j=1}^K \pi_k = 1\right\}$;
- state transition probability distribution, $\boldsymbol{A} = \left\{[a_{ij}] \mid \sum_{j=1}^K a_{ij},\ \forall i = 1, \ldots, K\right\}$;

- emission probability distribution $\boldsymbol{O}_t^s = \{\mathcal{O}_{kt}^s \mid \forall\, k = 1, \ldots, K\}$.

Given $\boldsymbol{A}$, $\boldsymbol{\pi}$, and using Markovian properties, the probability mass for the latent state variables is expressed as:

$$p(\underline{\boldsymbol{Z}} \mid \boldsymbol{\pi}, \boldsymbol{A}) = \prod_{s=1}^{S} p(\boldsymbol{z}_1^s \mid \boldsymbol{\pi}) \prod_{t=2}^{T} p(\boldsymbol{z}_t^s \mid \boldsymbol{z}_{t-1}^s, \boldsymbol{A}). \tag{1}$$

We assume that at a given latent state k in time t, shown by $z_{kt}^s = 1$, the observed vector $\boldsymbol{y}_t^s$ is generated via probabilistic interpretation of factor analysis model[8,9] as:

$$\boldsymbol{y}_t^s = \boldsymbol{U}_k \boldsymbol{x}_{kt}^s + \boldsymbol{\mu}_k + \boldsymbol{e}_{kt}, \quad \forall t, s \mid z_{kt}^s = 1,$$

where $\boldsymbol{U}_k = (\boldsymbol{u}_{k1}, \ldots, \boldsymbol{u}_{kP})$ is the linear transformation matrix, and P is the dimensionality of the latent space variable (in general $P < D$), $\boldsymbol{\mu}_k$ is the overall bias, $\boldsymbol{e}_{kt}(t)$ is the measurement noise. With the normality assumption, that is $\boldsymbol{x}_{kt}^s \sim \mathcal{N}(\boldsymbol{0}, 1)$ and $\boldsymbol{e}_{kt} \sim \mathcal{N}(\boldsymbol{0}, \boldsymbol{\Psi}_k)$, the marginal distribution of $\boldsymbol{y}_t^s$ follows a Gaussian distribution as[9]: $p(\boldsymbol{y}_t^s \mid \boldsymbol{\mu}_k, \boldsymbol{U}_k, \boldsymbol{\Psi}_k) = \mathcal{N}\left(\boldsymbol{\mu}_k, \boldsymbol{U}_k \boldsymbol{U}_k^\top + \boldsymbol{\Psi}_k\right)$. Note that here we have assumed noise with the same covariance across subjects.

We then define a dynamical process on the latent space variables[10] using an autoregressive (AR) model of order R as (**Supplementary Figure** 1):

$$\boldsymbol{x}_{kt}^s = \bar{\boldsymbol{X}}_{kt}^s \vec{\boldsymbol{V}}_k + \boldsymbol{\epsilon}_{kt}, \quad \forall t, s \mid z_{kt}^s = 1,$$

where $\vec{\boldsymbol{V}}_k$ is a P^2R-dimensional vector defined as:

$$\vec{\boldsymbol{V}}_k = \left(\boldsymbol{v}_{k1}, \boldsymbol{v}_{k2}, \ldots, \boldsymbol{v}_{kP}\right)^\top, \quad \text{where} \quad \boldsymbol{v}_{kp} = \begin{pmatrix} v_{kp,1} & v_{kp,2} & \ldots & v_{kp,PR} \end{pmatrix},$$

$\bar{\boldsymbol{X}}_{kt}^s$ is a $(P \times P^2R)$-dimensional matrix defined using a $(1 \times RP)$-dimensional vector $\bar{\boldsymbol{x}}_{kt}^s$ as:

$$\bar{\boldsymbol{X}}_{kt}^s = \begin{pmatrix} \bar{\boldsymbol{x}}_{kt}^s & \boldsymbol{0}_{RP} & \cdots & \boldsymbol{0}_{RP} \\ \boldsymbol{0}_{RP} & \bar{\boldsymbol{x}}_{kt}^s & \cdots & \boldsymbol{0}_{RP} \\ \vdots & \vdots & \ddots & \vdots \\ \boldsymbol{0}_{RP} & \boldsymbol{0}_{RP} & \cdots & \bar{\boldsymbol{x}}_{kt}^s \end{pmatrix}, \ \text{where} \ \bar{\boldsymbol{x}}_{kt}^s = \begin{pmatrix} {\boldsymbol{x}_{k,t-1}^s}^\top & {\boldsymbol{x}_{k,t-2}^s}^\top & \cdots & {\boldsymbol{x}_{k,t-R}^s}^\top \end{pmatrix},$$

where $\boldsymbol{0}_{RP}$ is a $(1 \times RP)$-dimensional zero-vector and $\boldsymbol{x}_{k,t-r}^s$ is a P-dimensional vector of latent space variables at $t - r$, and finally $\boldsymbol{\epsilon}_{kt} \sim \mathcal{N}(\boldsymbol{m}_k, \boldsymbol{\Sigma}_k)$ is the remaining error term in the latent space.

A graphical representation of the model is presented in **Supplementary Figure** 1. We have introduced some hierarchical parameters, ν_{kp}, which are not explicit in the generative model. As we shall see in § 1.2, these hyperparameters play the role of regularizers on the model complexity and are used to determine effective number of latent space variables. The idea of using such hierarchical structure was first introduced by[11,12] and it is now regarded as a common approach for regularizing model complexity by imposing sparsity in various Bayesian probabilistic models, for example, in Bayesian principal component analysis[13,14]. **Supplementary Table** 1 summarizes various variables introduced in this section.

Given the latent state variables $\boldsymbol{z}_t^s$, the marginal density of $\boldsymbol{y}_t^s$ is given by:

$$p(\boldsymbol{y}_t^s | \boldsymbol{z}_t^s, \boldsymbol{\mu}, \boldsymbol{U}, \boldsymbol{\Psi}) = \prod_{k=1}^{K} \int p(\boldsymbol{y}_t^s | \boldsymbol{x}_{kt}^s, \boldsymbol{z}_t^s, \boldsymbol{\mu}, \boldsymbol{U}, \boldsymbol{\Psi}) p(\boldsymbol{x}_{kt}^s) d\boldsymbol{x}_{kt}^s = \prod_{k=1}^{K} \left[\mathcal{N}\left(\boldsymbol{\mu}_k, \boldsymbol{U}_k \boldsymbol{U}_k^\top + \boldsymbol{\Psi}_k\right)\right]^{z_{kt}^s}, \tag{2}$$

where the effect of autoregressive process model parameters, $(\vec{\boldsymbol{V}},\boldsymbol{\Sigma},\boldsymbol{m})$, are implicit in the definition of the marginal density of $\boldsymbol{y}_t^s$. More specifically, $\boldsymbol{\Sigma}_k$ in Eq. (2) is assumed as identity matrix to avoid possible identifiability/degeneracy problems. Note that $\boldsymbol{\Sigma}_k$ includes information about the noise variance in the latent space variables. We will still need to formally compute its posterior distribution in order to robustly estimate parameters involved in the expression of the dynamical process on the latent space variables. However, on the observation level, the effect of $\boldsymbol{\Sigma}_k$ remains throughout as identity to avoid degeneracy.

To keep notation uncluttered, we define: $\boldsymbol{\theta}^{\text{HMM}}=\{\boldsymbol{\pi},\boldsymbol{A}\}$, $\boldsymbol{\theta}^{\text{FA}}=\{\boldsymbol{\mu},\boldsymbol{U},\boldsymbol{\Psi}\}$, and $\boldsymbol{\theta}^{\text{AR}}=\{\vec{\boldsymbol{V}},\boldsymbol{\Sigma},\boldsymbol{m}\}$. Note that $\boldsymbol{\theta}^{\text{HMM}}$, $\boldsymbol{\theta}^{\text{FA}}$, and $\boldsymbol{\theta}^{\text{AR}}$ are group parameters, learnt using observed data from all subjects. The latent state variables, $\boldsymbol{z}_t^s$, and latent space variables, $\boldsymbol{x}_{kt}^s$, are however subject specific, inferred using the learnt group parameters.

Using Eq. (1), and Eq. (2), the joint distribution over observations and latent state variables is given by:

$$p(\underline{\boldsymbol{Y}},\underline{\boldsymbol{Z}}|\underline{\boldsymbol{X}},\boldsymbol{\theta}^{\text{HMM}},\boldsymbol{\theta}^{\text{FA}})=\prod_{s=1}^{S}p(\boldsymbol{z}_1^s|\boldsymbol{\pi})\left[\prod_{t=2}^{T}p(\boldsymbol{z}_t^s|\boldsymbol{z}_{t-1}^s,\boldsymbol{A})\right]\left[\prod_{t=1}^{T}p(\boldsymbol{y}_t^s|\boldsymbol{z}_t^s,\boldsymbol{x}_{kt}^s,\boldsymbol{\theta}^{\text{FA}})\right]. \tag{3}$$

1.2 Prior parameter distribution

Bayesian inference combines priors with data to produce posterior distributions of all model parameters. Here, we work with conjugate priors to the data likelihood so that the posteriors would have the same functional form as their priors. This would simplify learning and allow efficient implementations.

1.2.1 Prior over HMM parameters

Following Bayesian HMM model[15], the prior distributions on the HMM model parameters are chosen as follows:

a. $p(\boldsymbol{\pi})=\text{Dir}(\alpha^*_{\boldsymbol{\pi}}\boldsymbol{m}^*), \quad \text{such that} \quad \boldsymbol{m}^*=\left[\frac{1}{K},\ldots,\frac{1}{K}\right]^\top,$

b. $p(\boldsymbol{A})=\prod_{i=1}^{K}p(\boldsymbol{a}_i)=\prod_{i=1}^{K}\text{Dir}(\alpha^*_{\boldsymbol{a}_i}\boldsymbol{m}^*), \quad \text{such that} \quad \boldsymbol{m}^*=\left[\frac{1}{K},\ldots,\frac{1}{K}\right]^\top,$

where, for instance, $\text{Dir}(\alpha^*_{\boldsymbol{\pi}}\boldsymbol{m}^*)$ is a symmetric Dirichlet distribution with strength $\alpha^*_{\boldsymbol{\pi}}$. $\boldsymbol{a}_i$ indicates the i-th row of the transition matrix $\boldsymbol{A}$. For a noninformative initialization, we set $\alpha^*_{\boldsymbol{\pi}}=\alpha^*_{\boldsymbol{a}_i}=1$, $\forall i=1,\ldots,K$.

1.2.2 Prior over factor analysis model parameters

We use the same choice of prior distributions over the factor analysis model parameters, $\boldsymbol{\theta}^{\text{FA}}$, as proposed by Ghahramani and Beal[14] as follows:

a. $p(\boldsymbol{U}|\boldsymbol{\nu}) = \prod_{k=1}^{K}\prod_{d=1}^{D} p(\dot{\boldsymbol{u}}_{kd}|\boldsymbol{\nu}_k) = \prod_{k=1}^{K}\prod_{d=1}^{D}\mathcal{N}\left(\mathbf{0}, \mathrm{diag}(\boldsymbol{\nu}_k)^{-1}\right)$, where $\dot{\boldsymbol{u}}_{kd}$ shows the column vector corresponding to the d-th row of the linear transformation matrix $\boldsymbol{U}$ at the k-th state and $\mathbf{0}$ is a P-dimensional vector of zero values.

b. $p(\boldsymbol{\nu}) = \prod_{k=1}^{K}\prod_{p=1}^{P} p(\nu_{kp}) = \prod_{k=1}^{K}\prod_{p=1}^{P}\mathcal{G}(a^*, b^*)$, where a^* and b^* are shape and inverse-scale hyperparameters for a Gamma distribution. For a noninformative initialization we set these parameters as: $a^* = b^* = 1$. As discussed in[14], when $\nu \rightarrow \inf$, then the outgoing weights for latent space variable $\boldsymbol{x}_{kt}^{s}$ will approach zero, allowing the model to reduce the intrinsic dimensionality of the latent space if data do not provide enough evidence to keep the added dimension—this effect is commonly known as automatic relevance determination in Bayesian learning[11,12,13].

c. $p(\boldsymbol{\mu}) = \prod_{k=1}^{K} p(\boldsymbol{\mu}_k) = \prod_{k=1}^{K}\mathcal{N}\left(\boldsymbol{\mu}^*, \mathrm{diag}(\boldsymbol{\nu}^*)^{-1}\right)$. For a noninformative initialization we set $\boldsymbol{\mu}^* = \mathbf{0}_D$, $\forall\ k = 1, \ldots, K$, where $\mathbf{0}_D$ is a D-dimensional zero vector and $\boldsymbol{\nu}^* = 10^{-3} \times \mathbf{1}_D$, where $\mathbf{1}_D$ is a D-dimensional vector of unit values.

1.2.3 Prior over autoregressive process model parameters

We use the same choice of prior distributions proposed by Fox[10], as:

a. $p(\vec{\boldsymbol{V}}) = \prod_{k=1}^{K} p(\vec{\boldsymbol{V}}_k) = \prod_{k=1}^{K}\mathcal{N}\left(\boldsymbol{m}_k^{(\vec{\boldsymbol{V}})*}, \boldsymbol{\Sigma}_k^{(\vec{\boldsymbol{V}})*}\right)$, where $\boldsymbol{m}^{(\vec{\boldsymbol{V}})*}$ is a P^2R-dimensional vector, and $\boldsymbol{\Sigma}_k^{(\vec{\boldsymbol{V}})*}$ is a $P^2R \times P^2R$-dimensional covariance matrix of the Gaussian densities.

b. $p(\boldsymbol{\Sigma}) = \prod_{k=1}^{K} p(\boldsymbol{\Sigma}_k) = \prod_{k=1}^{K}\mathcal{IW}\left(\nu_k^{(\boldsymbol{\Sigma})*}, \boldsymbol{S}_k^{(\boldsymbol{\Sigma})*}\right)$ where $\nu_k^{(\boldsymbol{\Sigma})*}$ and $\boldsymbol{S}_k^{(\boldsymbol{\Sigma})*}$ indicate the degree of freedom and scale matrix of the inverse-Wishart densities.

c. $p(\boldsymbol{m}) = \prod_{k=1}^{K} p(\boldsymbol{m}_k) = \prod_{k=1}^{K}\mathcal{N}\left(\boldsymbol{\mu}_k^{(\boldsymbol{m})*}, \boldsymbol{\Sigma}_k^{(\boldsymbol{m})*}\right)$ where $\boldsymbol{\mu}_k^{(\boldsymbol{m})*}$ and $\boldsymbol{\Sigma}_k^{(\boldsymbol{m})*}$ are mean vectors and covariance matrices of the Gaussian densities.

1.3 Variational inference

In a Bayesian view to uncertainty, all uncertain quantities are treated as random variables. Bayesian approach integrates over possible settings of all random variables. The resulting quantity is known as the marginal likelihood. Following the graphical model in **Supplementary Figure** 1, the marginal likelihood for the BSDS model is expressed as:

$$p(\underline{\boldsymbol{Y}}) = \prod_{s=1}^{S}\prod_{t=1}^{T}\sum_{k=1}^{K}\int\int\int p(\boldsymbol{y}_t^s, z_{kt}^s, \boldsymbol{x}_{kt}^s, \boldsymbol{\theta}_k^{\mathsf{FA}}, \boldsymbol{\theta}_k^{\mathsf{AR}}, \boldsymbol{\theta}_k^{\mathsf{HMM}})\ d\boldsymbol{x}_{kt}^s\ d\boldsymbol{\theta}_k^{\mathsf{FA}}\ d\boldsymbol{\theta}_k^{\mathsf{AR}}\ d\boldsymbol{\theta}_k^{\mathsf{HMM}},$$

where $p(\boldsymbol{y}_t^s, z_{kt}^s, \boldsymbol{x}_t^s, \boldsymbol{\theta}_k^{\mathsf{FA}}, \boldsymbol{\theta}_k^{\mathsf{AR}}, \boldsymbol{\theta}_k^{\mathsf{HMM}})$ is the joint probability distribution over all variables given by

$$p(\boldsymbol{y}_t^s, z_{kt}^s, \boldsymbol{x}_{kt}^s, \boldsymbol{\theta}_k^{\mathsf{FA}}, \boldsymbol{\theta}_k^{\mathsf{AR}}, \boldsymbol{\theta}_k^{\mathsf{HMM}}) = p(\boldsymbol{y}_t^s|\boldsymbol{z}_t^s, \boldsymbol{x}_t^s, \boldsymbol{\theta}_k^{\mathsf{FA}})p(\boldsymbol{x}_t^s|z_{kt}^s)p(z_{kt}^s|\boldsymbol{\theta}_k^{\mathsf{HMM}})p(\boldsymbol{\theta}_k^{\mathsf{HMM}})p(\boldsymbol{\theta}_k^{\mathsf{FA}})p(\boldsymbol{\theta}_k^{\mathsf{AR}}), \quad (4)$$

where $p(\boldsymbol{\theta}_k^{\mathsf{HMM}})$, $p(\boldsymbol{\theta}_k^{\mathsf{FA}})$, $p(\boldsymbol{\theta}_k^{\mathsf{AR}})$ are the prior distributions. The choice of prior distributions is discussed in § 1.2.

Given the observed data and the priors, the posterior distribution may be inferred using Bayes's rule as

$$p(\underline{\boldsymbol{Z}},\underline{\boldsymbol{X}},\boldsymbol{\theta}^{\mathsf{FA}},\boldsymbol{\theta}^{\mathsf{AR}},\boldsymbol{\theta}^{\mathsf{HMM}}|\underline{\boldsymbol{Y}})=\frac{p(\underline{\boldsymbol{Y}},\underline{\boldsymbol{Z}},\underline{\boldsymbol{X}},\boldsymbol{\theta}^{\mathsf{FA}},\boldsymbol{\theta}^{\mathsf{AR}},\boldsymbol{\theta}^{\mathsf{HMM}})}{p(\underline{\boldsymbol{Y}})}.$$

Directly computing the posterior may not be analytically tractable. In the following, we approximate the posterior using a family of optimization methods known as variational inference[16,17]. Variational inference is based on reformulating the problem of computing the posterior distribution as an optimization problem. Here, we work with a particular class of variational methods known as mean-field methods, which are based on optimizing Kullback-Leibler divergence[16]. We aim to minimize the Kullback-Leibler divergence between the variational posterior distribution, shown as $q(\underline{\boldsymbol{Z}},\underline{\boldsymbol{X}},\boldsymbol{\theta}^{\mathsf{FA}},\boldsymbol{\theta}^{\mathsf{AR}},\boldsymbol{\theta}^{\mathsf{HMM}})$, and the true posterior distribution, $p(\underline{\boldsymbol{Z}},\underline{\boldsymbol{X}},\boldsymbol{\theta}^{\mathsf{FA}},\boldsymbol{\theta}^{\mathsf{AR}},\boldsymbol{\theta}^{\mathsf{HMM}}|\underline{\boldsymbol{Y}})$, which can be expressed as:

$$\begin{aligned}\mathcal{D}\left[q(\underline{\boldsymbol{Z}},\underline{\boldsymbol{X}},\boldsymbol{\theta}^{\mathsf{FA}},\boldsymbol{\theta}^{\mathsf{AR}},\boldsymbol{\theta}^{\mathsf{HMM}})\;||\;p(\underline{\boldsymbol{Z}},\underline{\boldsymbol{X}},\boldsymbol{\theta}^{\mathsf{FA}},\boldsymbol{\theta}^{\mathsf{AR}},\boldsymbol{\theta}^{\mathsf{HMM}}|\underline{\boldsymbol{Y}})\right]=&\\ =-\left\langle \log\frac{p(\underline{\boldsymbol{Y}},\underline{\boldsymbol{Z}},\underline{\boldsymbol{X}},\boldsymbol{\theta}^{\mathsf{FA}},\boldsymbol{\theta}^{\mathsf{AR}},\boldsymbol{\theta}^{\mathsf{HMM}})}{q(\underline{\boldsymbol{Z}},\underline{\boldsymbol{X}},\boldsymbol{\theta}^{\mathsf{FA}},\boldsymbol{\theta}^{\mathsf{AR}},\boldsymbol{\theta}^{\mathsf{HMM}})}\right\rangle_{q(\underline{\boldsymbol{Z}},\underline{\boldsymbol{X}},\boldsymbol{\theta}^{\mathsf{FA}},\boldsymbol{\theta}^{\mathsf{AR}},\boldsymbol{\theta}^{\mathsf{HMM}})}&+\log p(\underline{\boldsymbol{Y}}),\end{aligned}\tag{5}$$

where the operator $\langle\cdot\rangle$ takes the expectation of variables in its argument with respect to the variational distribution $q(\cdot)$, e.g., $\langle f(x)\rangle_{g(x)}=\int f(x)g(x)dx$. The minimization of Eq. (5) is equivalent to the maximization of a *lower bound*, $\mathcal{L}$, on the log marginal likelihood defined as:

$$\mathcal{L}=\left\langle \log\frac{p(\underline{\boldsymbol{Y}},\underline{\boldsymbol{Z}},\underline{\boldsymbol{X}},\boldsymbol{\theta}^{\mathsf{FA}},\boldsymbol{\theta}^{\mathsf{AR}},\boldsymbol{\theta}^{\mathsf{HMM}})}{q(\underline{\boldsymbol{Z}},\underline{\boldsymbol{X}},\boldsymbol{\theta}^{\mathsf{FA}},\boldsymbol{\theta}^{\mathsf{AR}},\boldsymbol{\theta}^{\mathsf{HMM}})}\right\rangle_{q(\underline{\boldsymbol{Z}},\underline{\boldsymbol{X}},\boldsymbol{\theta}^{\mathsf{FA}},\boldsymbol{\theta}^{\mathsf{AR}},\boldsymbol{\theta}^{\mathsf{HMM}})}.\tag{6}$$

For the mean-field framework to yield a computationally efficient inference method, it is necessary to choose a family of distributions $q(\underline{\boldsymbol{Z}},\underline{\boldsymbol{X}},\boldsymbol{\theta}^{\mathsf{FA}},\boldsymbol{\theta}^{\mathsf{AR}},\boldsymbol{\theta}^{\mathsf{HMM}})$ such that we can tractably optimize the lower bound. Here, we approximate the true posterior distribution using a partly factorized approximation in the form of:

$$p(\underline{\boldsymbol{Z}},\underline{\boldsymbol{X}},\boldsymbol{\theta}^{\mathsf{FA}},\boldsymbol{\theta}^{\mathsf{AR}},\boldsymbol{\theta}^{\mathsf{HMM}})\simeq q(\underline{\boldsymbol{Z}},\underline{\boldsymbol{X}},\boldsymbol{\theta}^{\mathsf{FA}},\boldsymbol{\theta}^{\mathsf{AR}},\boldsymbol{\theta}^{\mathsf{HMM}})=q(\boldsymbol{\theta}^{\mathsf{FA}})q(\boldsymbol{\theta}^{\mathsf{AR}})q(\boldsymbol{\theta}^{\mathsf{HMM}})q(\underline{\boldsymbol{X}}|\underline{\boldsymbol{Z}})q(\underline{\boldsymbol{Z}}).\tag{7}$$

Given our choice of factorization in Eq. (7), we can express the lower bound explicitly as:

$$\mathcal{L}=\underbrace{\left\langle\left\langle \log\frac{p(\underline{\boldsymbol{Y}},\underline{\boldsymbol{X}},\boldsymbol{\theta}^{\mathsf{FA}},\boldsymbol{\theta}^{\mathsf{AR}}|\underline{\boldsymbol{Z}},\boldsymbol{\theta}^{\mathsf{HMM}})}{q(\underline{\boldsymbol{X}},\boldsymbol{\theta}^{\mathsf{FA}},\boldsymbol{\theta}^{\mathsf{AR}}|\underline{\boldsymbol{Z}})}\right\rangle_{q(\underline{\boldsymbol{X}},\boldsymbol{\theta}^{\mathsf{FA}},\boldsymbol{\theta}^{\mathsf{AR}}|\underline{\boldsymbol{Z}})}\right\rangle_{q(\underline{\boldsymbol{Z}})}}_{\mathcal{L}(\underline{\boldsymbol{Y}}|\underline{\boldsymbol{Z}})}+\left\langle\left\langle \log\frac{p(\underline{\boldsymbol{Z}},\boldsymbol{\theta}^{\mathsf{HMM}})}{q(\underline{\boldsymbol{Z}})q(\boldsymbol{\theta}^{\mathsf{HMM}})}\right\rangle_{q(\boldsymbol{\theta}^{\mathsf{HMM}})}\right\rangle_{q(\underline{\boldsymbol{Z}})},\tag{8}$$

where $\mathcal{L}$ has been separated into two parts. The first part, $\mathcal{L}(\underline{\boldsymbol{Y}}|\underline{\boldsymbol{Z}})$, is the $\underline{\boldsymbol{Z}}$-conditional expected value calculated using $q(\underline{\boldsymbol{X}},\boldsymbol{\theta}^{\mathsf{FA}},\boldsymbol{\theta}^{\mathsf{AR}}|\underline{\boldsymbol{Z}})$ across $\underline{\boldsymbol{X}}$, $\boldsymbol{\theta}^{\mathsf{FA}}$, $\boldsymbol{\theta}^{\mathsf{AR}}$ related only to the generation of $\underline{\boldsymbol{Y}}$. This part is calculated as a function of any given $\underline{\boldsymbol{Z}}$. The second term depends only on the Markov-chain model for the latent variables $\underline{\boldsymbol{Z}}$. Thus, the total lower bound can be maximized by alternating optimization of each of these two parts, while the other part of the model is kept unaltered.

1.4 Posterior parameter distribution

1.4.1 Posterior distributions of HMM parameters

For the model defined through the joint probability distribution in Eq. (4) and prior parameter distributions specified in $\S$ 1.2.1, posterior distributions of the HMM model parameters, $q(\boldsymbol{\theta}^{\text{HMM}})=q(\boldsymbol{\pi})q(\boldsymbol{A})$, which maximize the lower bound, Eq. (8), are given by:

a. a Dirichlet density in form of:

$$\begin{aligned} q(\boldsymbol{\pi}) &= \mathrm{Dir}(\boldsymbol{\alpha}_{\boldsymbol{\pi}}) \\ \boldsymbol{\alpha}_{\boldsymbol{\pi}} &= \sum_{s=1}^{S}(\alpha^*_{\boldsymbol{\pi}}\boldsymbol{m}^*) + \langle \boldsymbol{z}_1^s \rangle_{q(\boldsymbol{z}_t^s)} \end{aligned}, \tag{9}$$

b. and independent Dirichlet densities on each row of $\boldsymbol{A}$ in form of:

$$\begin{aligned} q(\boldsymbol{A}) &= \prod_{i=1}^{K}\boldsymbol{a}_i = \prod_{i=1}^{K}\mathrm{Dir}(\boldsymbol{\alpha}_{\boldsymbol{a}_i}) \\ \boldsymbol{\alpha}_{\boldsymbol{A}} &= \begin{bmatrix} \boldsymbol{\alpha}_{\boldsymbol{a}_1} \\ \boldsymbol{\alpha}_{\boldsymbol{a}_K} \end{bmatrix} = \begin{bmatrix} \alpha^*_{\boldsymbol{a}_1}\boldsymbol{m}^{*\top} \\ \alpha^*_{\boldsymbol{a}_K}\boldsymbol{m}^{*\top} \end{bmatrix} + \sum_{s=1}^{S}\sum_{t=2}^{T}\langle \boldsymbol{z}_{t-1}^s \ \boldsymbol{z}_t^s \rangle_{q(\boldsymbol{z}_t^s)} \end{aligned}. \tag{10}$$

Using Eq. (9) and Eq. (10), it follows:

$$\langle \log a_{ij} \rangle_{q(\boldsymbol{A})} = \psi(\alpha_{a_{ij}}) - \psi\left(\sum_{l=1}^{K}\alpha_{a_{i,l}}\right), \quad \forall\, i,j=1,\dots,K\ , \tag{11}$$

$$\langle \log \pi_k \rangle_{q(\boldsymbol{\pi})} = \psi(\alpha_{\pi_k}) - \psi\left(\sum_{j=1}^{K}\alpha_{\pi_j}\right), \quad \forall\, k=1,\dots,K\ , \tag{12}$$

where $\psi(\cdot)$ indicates the mathematical digamma function, $\alpha_{a_{ij}}$ is the j-th element of $\boldsymbol{\alpha}_{\boldsymbol{a}_i}$ and similarly α_{π_k} is the k-th element of $\boldsymbol{\alpha}_{\boldsymbol{\pi}}$.

1.4.2 Posterior distributions of factor analysis parameters

At a given latent state, $z_{kt}^s=1$, and a given latent space variable characterized with the sufficient statistics $\overline{\widetilde{\boldsymbol{x}}}_{kt}^s$ and $\overline{\widetilde{\boldsymbol{x}}_{kt}^s(\widetilde{\boldsymbol{x}}_{kt}^s)^\top}$, generative model of the BSDS can be viewed as a factor analysis model with an autoregressive process on its factor sources (latent space variables). Hence, in the following, we adopt similar methodology as in static factor analysis model[14]. For detailed discussions on the static factor analysis, readers are referred to[18].

For the model defined through the joint probability distribution in Eq. (4) and prior parameter distributions specified in $\S$ 1.2.2, posterior distributions of the model parameters which maximize the lower bound, Eq. (8), are given as follows:

a. Posterior distribution of $\boldsymbol{U}$ and $\boldsymbol{\mu}$ is given by independent Gaussian densities in form of:

$$q\left(\begin{bmatrix}\boldsymbol{U}\\ \boldsymbol{\mu}\end{bmatrix}\right)=\prod_{k=1}^{K}\prod_{d=1}^{D}q\left(\begin{bmatrix}\dot{\boldsymbol{u}}_{kd}\\ \mu_{kd}\end{bmatrix}\right)=\prod_{k=1}^{K}\prod_{d=1}^{D}\mathcal{N}\left(\begin{bmatrix}\bar{\dot{\boldsymbol{u}}}_{kd}\\ \bar{\mu}_{kd}\end{bmatrix},\Gamma_{kd}\right), \tag{13}$$

where $\bar{\mu}_{kd}$ denotes the d-th element of $\boldsymbol{\mu}_k$ and $\dot{\boldsymbol{u}}_{kd}$ denotes the column vector corresponding to the d-th row of $\boldsymbol{U}_k$, and

$$\begin{aligned}
\Gamma_{kd}&=\begin{bmatrix}\left(\Sigma_{kd}^{\boldsymbol{UU}}\right)^{-1} & \left(\Sigma_{kd}^{\boldsymbol{U\mu}}\right)^{-1}\\ \left(\Sigma_{kd}^{\boldsymbol{\mu U}}\right)^{-1} & \left(\Sigma_{kd}^{\boldsymbol{\mu\mu}}\right)^{-1}\end{bmatrix},\\
\left(\Sigma_{kd}^{\boldsymbol{UU}}\right)^{-1}&=\mathrm{diag}\left(\langle\boldsymbol{\nu}_k\rangle_{q(\boldsymbol{\nu}_k)}\right)+\Psi_{dd}^{-1}\sum_{s=1}^{S}\sum_{t=1}^{T}\overline{\widetilde{\boldsymbol{x}}_{kt}^{s}\left(\widetilde{\boldsymbol{x}}_{kt}^{s}\right)^{\top}}\\
\left(\Sigma_{kd}^{\boldsymbol{U\mu}}\right)^{-1}&=\left(\Sigma_{kd}^{\boldsymbol{\mu U}}\right)^{-1}=\Psi_{dd}^{-1}\sum_{s=1}^{S}\sum_{t=1}^{T}\overline{\widetilde{\boldsymbol{x}}}_{kt}^{s}\\
\left(\Sigma_{kd}^{\boldsymbol{\mu\mu}}\right)^{-1}&=\nu_d^{*}+\Psi_{dd}^{-1}\sum_{s=1}^{S}\sum_{t=1}^{T}\langle z_{kt}^{s}\rangle_{q(z_{kt}^{s})}\\
\bar{\dot{\boldsymbol{u}}}_{kd}&=[\Gamma_{kd}]_{\boldsymbol{UU}}\left(\Psi_{dd}^{-1}\sum_{s=1}^{S}\sum_{t=1}^{T}y_{dt}^{s}\overline{\widetilde{\boldsymbol{x}}}_{kt}^{s}\right)\\
\bar{\mu}_{kd}&=[\Gamma_{kd}]_{\boldsymbol{\mu\mu}}\left(\Psi_{dd}^{-1}\sum_{s=1}^{S}\sum_{t=1}^{T}\langle z_{kt}^{s}\rangle_{q(z_{kt}^{s})}y_{dt}^{s}+\nu_d^{*}\mu_d^{*}\right).
\end{aligned} \tag{14}$$

b. Posterior distribution of $\boldsymbol{\nu}$ is given by independent Gamma densities in form of:

$$q(\boldsymbol{\nu})=\prod_{k=1}^{K}\prod_{d=1}^{D}p(\nu_{kd})=\prod_{k=1}^{K}\prod_{d=1}^{D}\mathcal{G}(a_k,b_k), \tag{15}$$

where a_k and b_k are the shape and inverse scale posterior hyperparameters of the Gamma densities given by,

$$a_k=a^{*}+\frac{D}{2},\quad b_k=b^{*}+\frac{1}{2}\sum_{d=1}^{D}\left\langle\boldsymbol{u}_{kd}^{\top}\boldsymbol{u}_{kd}\right\rangle_{q(\boldsymbol{U})}. \tag{16}$$

1.4.3 Posterior distribution of the autoregressive process parameters

For the model defined through the joint probability distribution in Eq. (4) and prior parameter distributions specified in § 1.2.3, posterior distribution of the model parameters $(\vec{\boldsymbol{V}},\boldsymbol{\Sigma},\boldsymbol{m})$ which maximizes the lower bound, Eq. (8), are given as follows:

a. Posterior distribution of the autoregressive coefficients are given by independent Gaussian densities in form of

$$p(\vec{\boldsymbol{V}})=\prod_{k=1}^{K}p(\vec{\boldsymbol{V}}_k)=\prod_{k=1}^{K}\mathcal{N}\left(\boldsymbol{m}_k^{(\vec{\boldsymbol{V}})},\boldsymbol{\Sigma}_k^{(\vec{\boldsymbol{V}})}\right), \tag{17}$$

with posterior hyperparameters,

$$\begin{aligned}
\boldsymbol{m}_k^{(\vec{\boldsymbol{V}})} &= \left(\boldsymbol{\Sigma}_k^{(\vec{\boldsymbol{V}})*}\right)^{-1} \boldsymbol{m}_k^{(\vec{\boldsymbol{V}})*} + \sum_{s=1}^{S}\sum_{t=1}^{T} \widetilde{\overline{\boldsymbol{X}}}_{kt}^{s\;\top} \langle \boldsymbol{\Sigma}_k \rangle_{q(\boldsymbol{\Sigma}_k)}^{-1} \left(\widetilde{\boldsymbol{x}}_{kt}^s - \langle \boldsymbol{m}_k \rangle_{q(\boldsymbol{m}_k)}\right), \\
\boldsymbol{\Sigma}_k^{(\vec{\boldsymbol{V}})} &= \left(\boldsymbol{\Sigma}_k^{(\vec{\boldsymbol{V}})*}\right)^{-1} + \sum_{s=1}^{S}\sum_{t=1}^{T} \widetilde{\overline{\boldsymbol{X}}}_{kt}^{s\;\top} \langle \boldsymbol{\Sigma}_k \rangle_{q(\boldsymbol{\Sigma}_k)}^{-1} \widetilde{\overline{\boldsymbol{X}}}_{kt}^{s}.
\end{aligned} \tag{18}$$

b. Posterior distribution of the noise covariance matrices in the process, $\boldsymbol{\Sigma}$, are given by inverse-Wishart densities in form of:

$$q(\boldsymbol{\Sigma}) = \prod_{k=1}^{K} q(\boldsymbol{\Sigma}_k) = \mathcal{IW}\left(\nu_k^{(\boldsymbol{\Sigma})}, \boldsymbol{S}_k^{(\boldsymbol{\Sigma})}\right), \tag{19}$$

with degree of freedom, $\nu_k^{(\boldsymbol{\Sigma})}$, and scale matrix $\boldsymbol{S}_k^{(\boldsymbol{\Sigma})}$ as

$$\begin{aligned}
\nu_k^{(\boldsymbol{\Sigma})} &= \nu_k^{(\boldsymbol{\Sigma})*} + \sum_{s=1}^{S}\sum_{t=1}^{T} \langle z_{kt}^s \rangle_{q(z_{kt}^s)}, \\
\boldsymbol{S}_k^{(\boldsymbol{\Sigma})} &= \boldsymbol{S}_k^{(\boldsymbol{\Sigma})*} + \sum_{s=1}^{S}\sum_{t=1}^{T} \left(\widetilde{\boldsymbol{x}}_t^s - \widetilde{\overline{\boldsymbol{X}}}_{kt}^{s} \langle \vec{\boldsymbol{V}}_k \rangle_{q(\vec{\boldsymbol{V}}_k)} - \langle \boldsymbol{m}_k \rangle_{q(\boldsymbol{m}_k)}\right) \left(\widetilde{\boldsymbol{x}}_t^s - \widetilde{\overline{\boldsymbol{X}}}_{kt}^{s} \langle \vec{\boldsymbol{V}}_k \rangle_{q(\vec{\boldsymbol{V}}_k)} - \langle \boldsymbol{m}_k \rangle_{q(\boldsymbol{m}_k)}\right)^{\top}.
\end{aligned} \tag{20}$$

c. Posterior distribution of the noise mean vectors, $\boldsymbol{m}$, are given by Gaussian densities in form of:

$$p(\boldsymbol{m}) = \prod_{k=1}^{K} p(\boldsymbol{m}_k) = \prod_{k=1}^{K} \mathcal{N}\left(\boldsymbol{\mu}_k^{(\boldsymbol{m})}, \boldsymbol{\Sigma}_k^{(\boldsymbol{m})}\right), \tag{21}$$

with posterior hyperparameters $\boldsymbol{\mu}_k^{(\boldsymbol{m})}$ and $\boldsymbol{\Sigma}_k^{(\boldsymbol{m})}$ given by:

$$\begin{aligned}
\boldsymbol{\mu}_k^{(\boldsymbol{m})} &= \left(\boldsymbol{\Sigma}_k^{(\boldsymbol{m})*}\right)^{-1} \boldsymbol{\mu}_k^{(\boldsymbol{m})*} + \langle \boldsymbol{\Sigma}_k \rangle_{q(\boldsymbol{\Sigma}_k)}^{-1} \sum_{s=1}^{S}\sum_{t=1}^{T} \widetilde{\boldsymbol{x}}_t^s - \widetilde{\overline{\boldsymbol{X}}}_{kt}^{s} \langle \vec{\boldsymbol{V}}_k \rangle_{q(\vec{\boldsymbol{V}}_k)}, \\
\boldsymbol{\Sigma}_k^{(\boldsymbol{m})} &= \boldsymbol{\Sigma}_k^{(\boldsymbol{m})*} + \langle \boldsymbol{\Sigma}_k \rangle_{q(\boldsymbol{\Sigma}_k)}^{-1} \sum_{s=1}^{S}\sum_{t=1}^{T} \langle z_{kt}^s \rangle_{q(z_{kt}^s)}.
\end{aligned} \tag{22}$$

1.5 Optimization of the posterior hyperparameters

We then follow a similar approach as in static factor analysis model[14] for optimization of the posterior hyperparameters, $\{\alpha^* m^*, a^*, b^*, \boldsymbol{\mu}^*, \boldsymbol{\nu}^*, \boldsymbol{\Psi}\}$, in the model. The optimized hyperparameters are computed as follows:

a. Noise covariance, $\boldsymbol{\Psi}$, is updated using maximum likelihood estimation given by:

$$\boldsymbol{\Psi}^{-1} = \mathrm{diag}\left(\frac{1}{TS}\sum_{s=1}^{S}\sum_{t=1}^{T}\sum_{k=1}^{K} \left\langle \Phi\Phi^{\top} \right\rangle_{q\left(\begin{bmatrix}\boldsymbol{U}_k \\ \boldsymbol{\mu}_k\end{bmatrix}\right) q(\boldsymbol{x}_{kt}^s | z_{kt}^s) q(z_{kt}^s)}\right), \tag{23}$$

where $\Phi = \boldsymbol{y}_t^s - \begin{bmatrix} \boldsymbol{U}_k \\ \boldsymbol{\mu}_k \end{bmatrix} \begin{bmatrix} \boldsymbol{x}_{kt}^s \\ 1 \end{bmatrix}$.

b. Hyperparameters a^*, b^* are computed from the following fixed-point equations:

$$\psi(a^*) = \log(b^*) + \frac{1}{KP} \sum_{k=1}^{K} \sum_{p=1}^{P} \langle \log \nu_{kp} \rangle_{q(\nu_{kp})}, \tag{24}$$

$$b^{*-1} = \frac{1}{a^* KP} \sum_{k=1}^{K} \sum_{p=1}^{P} \langle \nu_{kp} \rangle_{q(\nu_{kp})}. \tag{25}$$

c. The scale prior $m_k^* = \frac{1}{K}$ is fixed and $\alpha_{\boldsymbol{\pi}}^* = \alpha_{\boldsymbol{a}_i}^* = \alpha^*$ are given for all $i = 1, \ldots, K$ as:

$$\psi(\alpha^*) - \psi(\frac{\alpha^*}{K}) = \frac{1}{K} \sum_{k=1}^{K} \psi(\alpha) - \psi(\alpha m_k). \tag{26}$$

d. $\boldsymbol{\mu}^*$ and $\boldsymbol{\nu}^*$ are optimized as:

$$\boldsymbol{\mu}^* = \frac{1}{K} \sum_{k=1}^{K} \langle \boldsymbol{\mu}_k \rangle_{q(\boldsymbol{\mu}_k)}, \tag{27}$$

$$\boldsymbol{\nu}^* = [\nu_1^*, \ldots, \nu_D^*], \quad \text{with} \quad \nu_d^* = \frac{1}{K} \sum_{k=1}^{K} \langle (\mu_{kd} - \mu_d^*)(\mu_{kd} - \mu_d^*) \rangle_{q(\boldsymbol{\mu}_k)}. \tag{28}$$

1.6 Sufficient statistics of the latent variables

We only require to know sufficient statistics of the latent space variables and the latent state variables which are inferred for each subject using group-level learnt posterior parameters in § 1.4.

1.6.1 Sufficient statistics of the latent space variables

For the model defined through the joint probability distribution in Eq. (4) and the marginal density of $\boldsymbol{y}_t^s$ given by Eq. (2), sufficient statistics of the latent space variables are inferred as:

$$\overline{\widetilde{\boldsymbol{x}}}_{kt}^s = \overline{\widetilde{\boldsymbol{X}}}_{kt}^s \left\langle \vec{\boldsymbol{V}}_k \right\rangle_{q(\boldsymbol{V}_k)} + \langle \boldsymbol{\Sigma}_k \rangle_{q(\boldsymbol{\Sigma}_k)}, \tag{29}$$

$$\overline{\widetilde{\boldsymbol{x}}_{kt}^s (\widetilde{\boldsymbol{x}}_{kt}^s)^\top} = \overline{\widetilde{\boldsymbol{X}}}_{kt}^s \left\langle \vec{\boldsymbol{V}}_k \vec{\boldsymbol{V}}_k^\top \right\rangle_{q(\vec{\boldsymbol{V}}_k)} \overline{\widetilde{\boldsymbol{X}}}_{kt}^{s\,\top} + 2 \overline{\widetilde{\boldsymbol{X}}}_{kt}^s \vec{\boldsymbol{V}}_k \langle \boldsymbol{\Sigma}_k \rangle_{q(\boldsymbol{\Sigma}_k)}^\top + \left\langle \boldsymbol{\Sigma}_k \boldsymbol{\Sigma}_k^\top \right\rangle_{q(\boldsymbol{\Sigma}_k)}, \tag{30}$$

where $\overline{\widetilde{\boldsymbol{X}}}_{kt}^s$ is expressed using $\widetilde{\boldsymbol{x}}_{kt}^s$ as:

$$\overline{\widetilde{\boldsymbol{X}}}_{kt}^s = \begin{pmatrix} \bar{\boldsymbol{x}}_{kt}^s & \boldsymbol{0}_{RP} & \cdots & \boldsymbol{0}_{RP} \\ \boldsymbol{0}_{RP} & \bar{\boldsymbol{x}}_{kt}^s & \cdots & \boldsymbol{0}_{RP} \\ \vdots & \vdots & \ddots & \vdots \\ \boldsymbol{0}_{RP} & \boldsymbol{0}_{RP} & \cdots & \bar{\boldsymbol{x}}_{kt}^s \end{pmatrix}, \quad \bar{\boldsymbol{x}}_{kt}^s = \left(\widetilde{\boldsymbol{x}}_{k,t-1}^{s\ \top} \quad \widetilde{\boldsymbol{x}}_{k,t-2}^{s\ \top} \quad \ldots \quad \widetilde{\boldsymbol{x}}_{k,t-R}^{s\ \top} \right),$$

where $\widetilde{\boldsymbol{x}}_t^s$ denote the data representations at time t for subject s computed as:

$$\widetilde{\boldsymbol{x}}_{kt}^s = \langle z_{kt}^s \rangle_{q(z_{kt}^s)} \langle \boldsymbol{x}_{kt}^s \rangle_{q(\boldsymbol{x}_{kt}^s | z_{kt}^s)},$$
$$\langle \boldsymbol{x}_{kt}^s \rangle_{q(\boldsymbol{x}_{kt}^s | z_{kt}^s)} = \left(\boldsymbol{I}_{P\times P} + \left\langle \boldsymbol{U}_k^\top \boldsymbol{\Psi}^{-1} \boldsymbol{U}_k \right\rangle_{q(\boldsymbol{U}_k)} \right)^{-1} \langle \boldsymbol{U}_k \rangle_{q(\boldsymbol{U}_k)}^\top \boldsymbol{\Psi}^{-1} \left(\boldsymbol{y}_t^s - \langle \boldsymbol{\mu}_k \rangle_{q(\boldsymbol{\mu}_k)} \right). \tag{31}$$

1.6.2 Sufficient statistics of the latent state variables

Posterior distribution of the latent state variables, $q(\underline{\boldsymbol{Z}})$, are obtained by maximizing the second term in the lower bound expression by Eq. (8), and using the Markov properties for $\underline{\boldsymbol{Z}}$. In practice, a variant of the *forward algorithm* and *backward algorithm* can be used to efficiently compute the necessary marginal probabilities, namely, $\langle \boldsymbol{z}_t^s \rangle_{q(\boldsymbol{z}_t^s)}$ and $\langle \boldsymbol{z}_t^s \; \boldsymbol{z}_{t-1}^s \rangle_{q(\boldsymbol{z}_t^s)}$. Computation of these statistics using the forward and backward algorithms requires the emission probability distribution, $\boldsymbol{O}_t^s = \{\mathcal{O}_{kt}^s | 1 \le k \le K\}$, which is given by:

$$\mathcal{O}_{kt}^s = \exp(\mathcal{L}(\boldsymbol{y}_t^s | z_{kt}^s = 1)), \quad \forall\, k,t,s \tag{32}$$

where

$$\mathcal{L}(\boldsymbol{y}_t^s | z_{kt}^s = 1) = \langle \log p(\boldsymbol{y}_t^s | z_{kt}^s, \boldsymbol{x}_{kt}^s, \boldsymbol{U}_k, \boldsymbol{\Psi}_k,) \rangle_{q(\boldsymbol{U}_k) q(\boldsymbol{x}_{kt}^s | z_{kt}^s)} - \langle \log q(\boldsymbol{x}_{kt}^s | z_{kt}^s) \rangle_{q(\boldsymbol{x}_{kt}^s | z_{kt}^s)} =$$
$$= \frac{1}{2} \log |\boldsymbol{\Sigma}_k| - \frac{1}{2} \mathrm{trace} \left(\Psi^{-1} \left\langle \left(\boldsymbol{y}_t^s - \begin{bmatrix} \boldsymbol{U}_k \\ \boldsymbol{\mu}_k \end{bmatrix} \begin{bmatrix} \boldsymbol{x}_{kt}^s \\ 1 \end{bmatrix} \right) \left(\boldsymbol{y}_t^s - \begin{bmatrix} \boldsymbol{U}_k \\ \boldsymbol{\mu}_k \end{bmatrix} \begin{bmatrix} \boldsymbol{x}_{kt}^s \\ 1 \end{bmatrix} \right)^\top \right\rangle_{q\left(\begin{bmatrix} \boldsymbol{U}_k \\ \boldsymbol{\mu}_k \end{bmatrix} \right) q(\boldsymbol{x}_{kt}^s | z_{kt}^s)} \right). \tag{33}$$

Details of the forward-backward computations are discussed in the following.

Forward Algorithm The forward procedure is initialized for $t=1$ as

$$\alpha_{i1}^s = \tilde{a}_{Ki} \mathcal{O}_{it}^s, \quad \text{where } \tilde{a}_{ij} = \exp\left(\langle \log a_{ij} \rangle_{q(\boldsymbol{A})} \right), \quad \forall\, 1 \le i \le K.$$

Then, the forward iteration can formally run for $t = 2, \dots, T$ as

$$\alpha_{jt}^s = \sum_{i=1}^K \alpha_{t-1,i}^s \tilde{a}_{ij} \mathcal{O}_{jt}^s, \quad \forall\, 1 \le j \le K.$$

For later use in the computation of the lower bound, we compute the the normalization constant C as

$$C = \sum_{s=1}^S \sum_{k=1}^K \alpha_{kT}^s. \tag{34}$$

Backward Algorithm To supplement the forward calculation, we need a backward variable to represent the observed conditions after time t. The backward variable is initialized as $\beta_{jT}^s = 1$ for all $j = 1, \dots, K$. The backward variable is recursively defined for $t = T-1, \dots, 1$ as

$$\beta_{it}^s = \sum_{j=1}^K \tilde{a}_{ij} \mathcal{O}_{j,t+1}^s \beta_{j,t+1}^s, \quad \forall 1 \le i \le K. \tag{35}$$

Finally, $\langle z_{it}^s \rangle_{q(z_{it}^s)}$ and $\left\langle z_{it}^s z_{j,t-1}^s \right\rangle_{q(\boldsymbol{z}_t^s)}$ are given by:

$$\langle z_{it}^s \rangle_{q(z_{it}^s)} = \frac{\alpha_{it}^s \ \beta_{it}^s}{\sum_{k=1}^K \alpha_{kt}^s \ \beta_{kt}^s}, \tag{36}$$

$$\left\langle z_{it}^s z_{j,t-1}^s \right\rangle_{q(\boldsymbol{z}_t^s)} = \frac{\alpha_{j,t-1}^s \tilde{a}_{ij} \mathcal{O}_{it}^s \beta_{it}^s}{\sum_{k=1}^K \sum_{l=1}^K \alpha_{l,t-1}^s \tilde{a}_{kl} \mathcal{O}_{kt}^s \beta_{kt}^s}, \quad \forall\, 1 \le i,j \le K. \tag{37}$$

1.7 Algorithm

An algorithmic summary of BSDS is shown in Algorithm 1, **Supplementary Table** 2.

2 Measures

Transition probability between states Transition probabilities between latent states are given by:

$$\hat{\boldsymbol{A}} = [\hat{a}_{ij}] \quad \text{where} \quad \hat{a}_{ij} = \exp\Big(\langle \log a_{ij} \rangle_{q(\boldsymbol{A})}\Big), \quad \forall\, i,j = 1,\dots,K \tag{38}$$

where $\langle \log a_{ij} \rangle_{q(\boldsymbol{A})}$ is given by Eq. (11). Diagonal values on $\hat{\boldsymbol{A}}$ give the self-transition probabilities and off-diagonal values give the cross-transition probabilities.

Temporal evolution of states Temporal evolution of the latent states indicates to which latent state a given time point belongs and it is given by the *Viterbi path* defined as the most likely sequence of latent states in the sequence of observed data and is computed using Viterbi algorithm[2]. Explicitly, the temporal evolution of states for a given subject s is expressed by the Viterbi path and is computed using estimated output probability distribution $\mathcal{O}_{kt}^s$, given by Eq. (32), and the estimated transition probabilities given by Eq. (38). Note that, as the model parameters, ($\boldsymbol{\theta}^{\text{FA}}, \boldsymbol{\theta}^{\text{HMM}}, \boldsymbol{\theta}^{\text{AR}}$), are learnt in a group-level fashion using sufficient statistics from all subjects, there is a one-to-one correspondence between states across subjects such that a given state i would correspond to the same state for all subjects, $s = 1,\dots,S$.

Occupancy rate and mean lifetime of states The occupancy rate for state i and subject s is computed as:

$$\text{Occupancy Rate}(i,s) = \frac{\sum_{t=1}^T \delta(z_{it}^s = 1)}{T} \times 100, \tag{39}$$

where $\delta(z_{it}^s = 1)$ is the Dirac delta function which is one if the current state at time t is the i-th state. To know at which state we are at a given time, we will use the temporal evolution of states.

The mean lifetime of a state is the average time that a given state i continuously persists before switching to another state. This quantity can be simply computed from the temporal evolution of states.

Occupancy rate and mean life time are complementary of each other. These quantities are computed for all states and across all subjects. As there is a one-to-one correspondence between states across subjects, we can monitor their variations across subjects.

Group-level mean and covariance Using estimated posterior distribution of the model parameters, estimated group-level mean and covariance for each state are given by:

$$\mathrm{mean}_k = \bar{\boldsymbol{\mu}}_k, \quad \bar{\boldsymbol{\mu}}_k = [\bar{\mu}_{kd}, \forall\, d=1,\ldots,D], \quad \forall k=1,\ldots,K \tag{40}$$

$$\mathrm{cov}_k = \left(\bar{\boldsymbol{u}}_k \bar{\boldsymbol{u}}_k^\top\right) + \boldsymbol{\Psi}^{-1}, \quad \bar{\boldsymbol{u}}_k = [\bar{\boldsymbol{u}}_{kd}, \forall\, d=1,\ldots,D], \quad \forall k=1,\ldots,K \tag{41}$$

where $\bar{\mu}_{kd}$, $\bar{\boldsymbol{u}}_{kd}$ and $\boldsymbol{\Psi}^{-1}$ are given by Eq. (14) and Eq. (23). Note that, using temporal evolution of states and estimated covariance of states, we can now compute changes in covariance over time.

3 Subject-level learning using BSDS

As before, let $\{\boldsymbol{\theta}^{\mathsf{FA}}, \boldsymbol{\theta}^{\mathsf{HMM}}, \boldsymbol{\theta}^{\mathsf{AR}} | \boldsymbol{\theta}_k^{\mathsf{FA}}, \boldsymbol{\theta}_k^{\mathsf{HMM}}, \boldsymbol{\theta}_k^{\mathsf{AR}}, \forall k=1,\ldots,K\}$ show the group level model parameters computed using data measurements from all subjects, $\underline{\boldsymbol{Y}} = \{\boldsymbol{Y}^s | s=1,\ldots,S\}$. To obtain subject level model parameters, shown as $\{\{\boldsymbol{\theta}^{\mathsf{FA}}\}^s, \{\boldsymbol{\theta}^{\mathsf{HMM}}\}^s, \{\boldsymbol{\theta}^{\mathsf{AR}}\}^s | \forall s=1,\ldots,S\}$, we initialize BSDS informative using sufficient statistics of the latent state variables computed from the group-level analysis, $\langle z_{kt}^s \rangle_{q(z_{kt}^s)}$. From the algorithmic point of view, we only need to replace initialization of $\langle \boldsymbol{z}_t^s \rangle_{q(\boldsymbol{z}_t^s)}$ in Step 1 of Algorithm 1 (**Supplementary Table** 2) by the estimated $\langle z_{kt}^s \rangle_{q(z_{kt}^s)}, \forall k$ from the group level analysis. BSDS then uses the prior and data from the given subject s to learn the subject-level posterior parameters $\{q(\boldsymbol{\theta}_k^{\mathsf{FA}})\}^s, \{q(\boldsymbol{\theta}_k^{\mathsf{AR}})\}^s, \{q(\boldsymbol{\theta}_k^{\mathsf{HMM}})\}^s$. A summary of algorithm is shown in Algorithm 2, **Supplementary Table** 3.

Subject-level mean and covariance Using estimated subject-level posterior distribution of the model parameters, $\{q(\boldsymbol{\theta}_k^{\mathsf{FA}})\}^s, \{q(\boldsymbol{\theta}_k^{\mathsf{AR}})\}^s, \{q(\boldsymbol{\theta}_k^{\mathsf{HMM}})\}^s, \forall s=1,\ldots,S$, the estimated subject-level mean and covariance matrices are given by Eq. (40) and Eq. (41).

Supplementary Note 1: Supplementary Results

1 Robustness of findings with respect to ROI selection: BSDS applied to HCP n-back working memory (WM) task with ROIs from ICA-derived resting-state brain networks

Our primary analysis focused on fronto-parietal ROIs based on peaks of task-related activation and deactivation. To examine the robustness of our findings with respect to ROI selection, we conducted a complete set of parallel supplemental analyses using functional clusters from previously published brain networks derived using ICA on resting-state fMRI[19]. Networks of interest included the salience network (SN), central executive network (CEN), default mode network (DMN) and dorsal attention network (DAN), from which we chose the following fronto-parietal regions: bilateral AI, bilateral DLPFC, bilateral FEF, bilateral PPC, PCC, VMPFC and right DMPFC (**Supplementary Figure** 5). All other processing steps were identical to the previous analysis. As described in detail below, all major findings were replicated with this more general resting-state network-derived choice of ROIs.

1.1 Matching BSDS states between sessions

We applied BSDS to probe latent brain dynamics associated with ROIs in two data sessions separately. BSDS isolated five latent brain states in Sessions 1 and 2. To determine whether one brain state identified in one session match one brain state identified in another session, we conducted cross-session brain state correlation analysis. Using the same state matching algorithm applied in the main analysis (see Material and Methods for details), we found exclusive one-to-one mapping of the five latent brain states between two sessions. The Pearson's correlation coefficients between the matched brain states across two sessions range from 0.56 to 0.87 (**Supplementary Table** 15).

1.2 Fractional occupancy of task-dominant latent brain states during WM task

The 2-back, 0-back and fixation conditions were each dominated by distinct brain states designated SH (high-load state), SL (low-load state), and SF (fixation state) respectively (**Supplementary Figure** 6). The brain state SH during the 2-back WM task had an occupancy rate of $47.1\pm6.6\%$ in Session 1 and $36\pm11.5\%$ in Session 2 (**Supplementary Figure** 6d). In both Sessions, the occurrence of the SH during the 2-back task condition was significantly higher than the occurrence of other non-dominant states (all p-values <0.001, two-tailed t-test) except the 5th State in Session 2. Although the 5th State had high occupancy rate in 2-back WM task in Session 2, it also had high occupancy rate in 0-back WM task but low occupancy rate in rest. Therefore, the 5th State did not have uniquely significant contribution to the 2-back task. So this state is designated SG (general-control state). The mean lifetime of the state SH in the 2-back condition was 13 ± 5 seconds in Session 1 and 9 ± 4 seconds in Session 2, significantly longer than the mean lifetime of the other brain states (all p-values <0.001, two-tailed t-test), but much shorter than the 27.5 seconds task block (**Supplementary Figure** 6e). Thus, the 2-back condition is characterized by a mixture of brain states, with the dominant state active for only a relatively short interval. A similar pattern was observed for the states that were dominant in the 0-back and

fixation conditions (**Supplementary Figure** 6d, 6e). These results demonstrate that the WM task is characterized by latent task-induced states whose fractional occupancy is relatively short compared to the task blocks.

1.3 Identification of a novel transition state

In addition to SH, SL, SF and SG, BSDS uncovered a transition state (ST in **Supplementary Figure** 6). The occupancy rate and mean lifetime of this state during the 2-back, 0-back and fixation conditions was comparably lower than the other states. However, ST was more likely to occur during transition after the onset of new task blocks ($27 \pm 19\%$ in Session 1 and $41 \pm 13\%$ in Session 2, **Supplementary Figure** 6f), significantly higher than other latent states in both sessions (p-values <0.05, two-tailed t-test) but not to SG in Session 1. These results demonstrate that cognitive tasks with multiple conditions are characterized not only by latent task-induced states but also by transition states.

1.4 Latent brain states predict WM performance

To probe the relation between latent brain states and task performance, we took advantage of a key feature of BSDS, which provides estimates of moment-by-moment changes in brain states and connectivity. We examined whether time-varying brain state changes could predict WM performance. Specifically, we trained a multiple linear regression model to fit estimated the 2-back accuracy using occupancy rates of brain states in the 2-back condition, applied the model on unseen data to predict accuracy, and evaluated model performance by comparing estimated accuracy and observed value across all the subjects. This analysis revealed a significant relation between predicted and actual accuracy (all p-values <0.005, Pearson's correlation, **Supplementary Figure** 7a). Notably, each of these results was replicated in both Sessions 1 and 2, highlighting the robustness of our brain-behavior findings.

We then tested the hypothesis that the occupancy rate of individual brain states in the 2-back condition is associated with performance in the 2-back block. We found that WM task accuracy was positively correlated with the occupancy rate of the dominant state, SH, in the 2-back WM task condition (all p-values <0.01, Pearson's correlation, **Supplementary Figure** 7b). Thus, the dominant state SH is a behaviorally optimal brain state for 2-back working performance the more time spent in this brain state the better WM task performance, with more deviations leading to poorer performance.

Next, we investigated the mean lifetime, another key feature of temporal evolution of latent brain states, in relation to WM performance using the same analytic procedures described above. We found that the mean lifetimes of the latent brain states in the 2-back condition predicted WM task accuracy (all p-values <0.05, Pearson's correlation, **Supplementary Figure** 7c). Thus, maintenance of optimal hidden brain states results in better WM task performance.

2 Robustness of findings with respect to head movement: BSDS applied to HCP n-back WM task with 12 head motion regression parameters

To examine whether the key latent brain states uncovered by BSDS are stable with respect to different motion correction procedures, we conducted additional analysis by regressing out 12 motion parameters, including 6 standard parameters (3 translational and 3 rotational movement) and derivatives of these 6 standard parameters, and then repeated the same BSDS analysis using 11 ROIs defined by 2-back vs 0-back contrast. As described in detail below, all major findings were replicated with this choice of movement parameters.

2.1 Matching BSDS states between sessions

We applied BSDS to probe latent brain dynamics associated with ROIs in two data sessions separately. BSDS isolated five latent brain states in Session 1 and Session 2. To determine whether one brain state identified in one session match one brain state identified in another session, we conducted cross-session brain state correlation analysis. Using the same state matching algorithm applied in the main analysis (see Material and Methods for details), we found exclusive one-to-one mapping of the five latent brain states between two sessions. The Pearson's correlation coefficients between the matched brain states across two sessions range from 0.93 to 0.97 (**Supplementary Table** 16).

2.2 Fractional occupancy of task-dominant latent brain states during WM task

The 2-back, 0-back and fixation conditions were each dominated by distinct brain states designated SH (high-load state), SL (low-load state), and SF (fixation state) respectively (**Supplementary Figure** 8). The brain state SH during the 2-back WM task had an occupancy rate of $37.6{\pm}12.2\%$ in Session 1 and $34.7{\pm}11.5\%$ in Session 2 (**Supplementary Figure** 8d). In both Sessions, the occurrence of the SH during the 2-back task condition was significantly higher than the occurrence of other non-dominant states (all p-values <0.001, two-tailed t-test) except the 5th State. Although the 5th State had high occupancy rate in 2-back WM task too, it also had high occupancy rate in 0-back WM task but low occupancy rate in rest. Therefore, the 5th State did not have uniquely significant contribution to the 2-back task. So this state is designated SG (general-control state). The mean lifetime of the state SH in the 2-back condition was $10{\pm}4$ seconds in Session 1 and $8{\pm}7$ seconds in Session 2, significantly longer than the mean lifetime of the other brain states (all p-values <0.001, two-tailed t-test), but much shorter than the 27.5 seconds task block (**Supplementary Figure** 8e). Thus, the 2-back condition is characterized by a mixture of brain states, with the dominant state active for only a relatively short interval. A similar pattern was observed for the states that were dominant in the 0-back and fixation conditions (**Supplementary Figure** 8d, 8e). These results demonstrate that the WM task is characterized by latent task-induced states whose fractional occupancy is relatively short compared to the task blocks.

2.3 Identification of a novel transition state

In addition to SH, SL, SF and SG, BSDS uncovered a transition state (ST in **Supplementary Figure** 8). The occupancy rate and mean lifetime of this state during the 2-back, 0-back and fixation conditions was comparably lower than the other states. However, ST was more likely to occur during transition after the onset of new task blocks ($30{\pm}20\%$ in Session 1 and $33{\pm}17\%$ in Session 2, **Supplementary Figure** 8f), significantly higher than other latent states in both sessions (p-values <0.05, two-tailed t-test) except that it is marginally significant compared to SH in Session 1 ($p{=}0.07$, two-tailed t-test). These results demonstrate that cognitive tasks with multiple conditions are characterized not only by latent task-induced states but also by transition states.

2.4 Latent brain states predict WM performance

To probe the relation between latent brain states and task performance, we took advantage of a key feature of BSDS, which provides estimates of moment-by-moment changes in brain states and connectivity. We examined whether time-varying brain state changes could predict WM performance. Specifically, we trained a multiple linear regression model to fit estimated the 2-back accuracy using occupancy rates of brain states in the 2-back condition, applied the model on unseen data to predict accuracy, and evaluated model performance by comparing estimated accuracy and observed value across all the subjects. This analysis revealed a significant relation between predicted and actual accuracy (all p-values <0.01, Pearson's correlation, **Supplementary Figure** 9a). Notably, each of these results was replicated in both Sessions 1 and 2, highlighting the robustness of our brain-behavior findings.

We then tested the hypothesis that the occupancy rate of individual brain states in the 2-back condition is associated with performance in the 2-back block. We found that WM task accuracy was positively correlated with the occupancy rate of the dominant state, SH, in the 2-back WM task condition (all p-values <0.005, Pearson's correlation, **Supplementary Figure** 9b). Conversely, the occupancy rate of non-dominant brain states during the 2-back task was associated with poorer performance. Thus, the dominant state SH is a behaviorally optimal brain state for 2-back working performance – the more time spent in this brain state the better WM task performance, with more deviations leading to poorer performance.

Next, we investigated the mean lifetime, another key feature of temporal evolution of latent brain states, in relation to WM performance using the same analytic procedures described above. We found that the mean lifetimes of the latent brain states in the 2-back condition predicted WM task accuracy (all p-values <0.01, Pearson's correlation, **Supplementary Figure** 9c). Thus, maintenance of optimal hidden brain states results in better WM task performance.

3 Performance of related HMM-based models on optofMRI and n-back WM data

We next examined the performance of a broad class of HMM based methods and applied them to opto-fMRI and n-back WM fMRI task data. HMM models can be categorized based on their inference into maximum-likelihood and Bayesian models. In its standard form, the ML-based

HMM model has limited application in practice, as the number of states has to be specified in advance. Thus, here, we only consider Bayesian HMM-based methods. The Bayesian models may also be broadly categorized into parametric Bayesian models and nonparametric Bayesian models. Bayesian inference in either forms provides a structured way of handling model complexity, and with sufficient amount of data, they can automatically prune away states with little influence. Here, we consider examples of each category.

In the following, we consider multiple Bayesian HMM-based methods and evaluate their performance on opto-fMRI and n-back WM data. Briefly, our analysis of these methods revealed they are unable to properly handle the model complexity by either overestimating uncertainties resulting in over-pruning of the latent states and converging to a single state, or underestimating uncertainties resulting in multiple states (>10) with little resemblance to the task. The latter case arises when models have limited flexibility and are strongly constrained by the Markovian assumptions as the only mechanism for capturing complex dynamical processes hidden in the data. Such models cannot deal with abrupt changes in data which may not be relevant to group patterns of interest. The former case arises in models where each state has extensive degree of flexibility with many parameters to be learnt from data. Although theoretically appealing, in practice, it is difficult to robustly learn all these parameters from noisy data and hence the model behaves too conservatively and can reliably estimate only one state.

In comparison, BSDS takes advantage of the strengths of each while minimizing the weaknesses. Similar to the nonparametric Bayesian switching linear dynamical systems[20], it is rich in flexibility. It benefits from both Markovian assumptions and the autoregressive processes in its latent space. However, its flexibility is constrained by using a parametric model with fewer parameters to estimate from the data. On the other hand, it is flexible enough to not react too quickly to every local change in data, allowing it to estimate an optimal and parsimonious set of latent states across study participants.

3.1 Related HMM-based methods

We considered the following HMM-based methods[21,20,22]:

Category 1 Hierarchical Dirichlet process hidden Markov model (HDP-HMM);

Category 2 Hierarchical Dirichlet process autoregressive hidden Markov model (HDP-AR-HMM);

Category 3 Hierarchical Dirichlet process switching linear dynamical systems (HDP-SLDS);

Category 4 Bayesian switching factor analysis (BSFA).

HDP-HMM belongs to the family of nonparametric Bayesian models. Seen as the simplest model, HDP-HMM captures temporal dependencies using HMM's Markovian assumptions. HDP-AR-HMM can be seen as a representative of another family of methods for modeling temporal data which uses autoregressive (AR) process within the HMM framework. When compared to the HDP-HMMs, HDP-AR-HMMs are more capable of capturing complex dynamical processes in data due to the additional use of AR in their generative model. BSFA can be seen as the simplest model which takes the advantage of latent space variables in the HMM framework. HDP-SLDS belongs to the same family of methods to which BSDS belongs.

HDP-SLDS is the most flexible model: It shares similarities with BSFA in the use of latent space variables, and can be seen as the general form of HDP-AR-HMM where AR process is defined on the latent space variables.

Categories 1-3 are based on Emily Fox toolbox, HDPHMM-HDPSLDS toolbox[1]. This toolbox implements various HDP-based models[21,20]. Category 4, BSFA[22], can be seen as a special case of BSDS without autoregressive process[2].

HDP-HMM/HDP-AR-HMM/HDP-SLDS supports time series analysis with various model families. We considered the following model families in our analysis (see HDPHMM-HDPSLDS toolbox for implementation details and refer to[21,20] for theoretical discussion):

<u>HDP-HMM</u>

HDP-HMM.A Gaussian emissions with non-conjugate Normal-inverse Wishart observation model type shown as N-IW prior.

HDP-HMM.B Gaussian emissions with conjugate Normal-inverse Wishart observation model type, shown as NIW prior.

<u>HDP-AR-HMM</u>

HDP-AR-HMM.A AR order fixed, conjugate matrix-Normal inverse-Wishart observation model type shown as MNIW prior.

HDP-AR-HMM.B Latent AR order with maximal order r with Normal prior together with conjugate matrix-Normal inverse-Wishart Normal observation model type shown as MNIW-N prior.

HDP-AR-HMM.C Latent AR order with maximal order r, Normal prior together with non-conjugate Normal inverse-Wishart observation model type shown as N-IW-N prior.

HDP-AR-HMM.D Latent AR order with maximal order r, automatic relevance determination observation model type shown as ARD prior.

<u>HDP-SLDS</u>

HDP-SLDS.A SLDS with conjugate matrix-Normal inverse-Wishart (MNIW) observation model type, and inverse Wishart (IW) prior on noise.

HDP-SLDS.B SLDS with conjugate matrix-Normal inverse-Wishart (MNIW) observation model type, and non-conjugate inverse Wishart Normal (IW-N) prior on noise.

HDP-SLDS.C SLDS with conjugate matrix-Normal inverse-Wishart (MNIW) observation model type, and conjugate inverse Normal Wishart Normal (NIW) prior on noise.

HDP-SLDS.D SLDS with non-conjugate Normal inverse-Wishart Normal (N-IW-N) observation model type, and inverse Wishart (IW) prior on noise.

HDP-SLDS.E SLDS with non-conjugate Normal inverse-Wishart Normal (N-IW-N) observation model type, and non-conjugate inverse Wishart Normal (IW-N) prior on noise.

[1]https://homes.cs.washington.edu/ ebfox/software/
[2]https://github.com/StanfordCosyne/BSFA

3.2 Analysis of opto-fMRI data

We applied all methods to opto-fMRI dataset to validate their performance on a real dataset for which the ground-truth is known to some extent. Only three of these methods could find more than one state, namely HDP-HMM.A,B, BSFA (**Supplementary Figure** 10-12). Both HDP-AR-HMM.A-D and HDP-SLD.A-E converged to only a single state.

HDP-HMM.A-B can be seen as a nonparametric variant of the HMM with Gaussian emission probability distributions. The key difference between HDP-HMM.A and HDP-HMM.B is in their choice of priors. While HDP-HMM.A uses non-conjugate prior model within a fully factorized prior model, HDP-HMM.B uses a conjugate prior model to avoid the full factorization of the priors which theoretically should help with the better recovery of states. Both methods capture temporal correlations in data using Markovian assumptions within an HDP framework. HDP-HMM.A converged to 4 states and HDP-HMM.B converged to 3 states. We also observed in our analysis that while both methods are rather sensitive to the random initialization, HDP-HMM.A appears to be slightly more sensitive in comparison to HDP-HMM.B.

BSFA is a state-space model which uses HMM to capture temporal dependencies as opposed to BSDS which uses both HMM and AR process wrapped into a state-space generative model. As shown in **Supplementary Figure** 12, the learning converges to several states (14 states) with no clear pattern. HDP-AR-HMM.A-D use both HMM and AR process in their generative models. We varied the AR order from 1 to 3. All methods consistently converged to a single state (results not shown here). HDP-SLDS.A-E are closest to the BSDS in their generative form but they differ in their inference. While BSDS uses variational inference, they use MCMC sampling. Furthermore, BSDS is a Bayesian parametric model while HDP-SLDS.A-E are Bayesian non-parametric models. In our analysis, we observed all cases converged to a single state.

3.3 Analysis of HCP WM data

As in the case of opto-fMRI data, HDP-AR-HMM and HDP-SLDS when applied to the WM dataset, converged to a single state.

HDP-HMM.A,B were initialized as in the case of validation dataset. Estimated temporal evolution of states is shown in **Supplementary Figures** 13, 14. HDP-HMM.A in Session 1 and 2 converged to 10 and 9 states respectively. Similarly, HDP-HMM.B converged to 10 states in Session 1 and 10 in Session 2. BSFA was initialized as in the case of validation dataset. Estimated temporal evolution of states is shown in **Supplementary Figure** 15. BSFA in Session 1 and 2 converged to 15 and 16 states, respectively.

3.4 General considerations

Within the HMM framework, another family of methods for modeling temporal data uses autoregressive (AR) process. In such models, each state of the HMM is assumed to be generated from an AR process. In a more general form, switching linear dynamical models are another important family of models for modeling timeseries. It should be noted that while prior studies have used HMMs for estimation of brain states on the observed data, unlike the present

study, they do not explicitly model latent processes underlying observed data. When compared to standard HMM-based models[21,23,24,22], crucially, BSDS applies HMM on the latent space variables, and it is their combination that creates a switching dynamical system which then generates the observed data. This is in contrast to previous approaches that have applied HMMs directly to observed MEG[24] and resting-state fMRI[23,22]. In comparison, each latent state of BSDS is richer and has greater flexibility due to the autoregressive processes in the latent space variables. Critically, the system only switches to a new state if the current state is not capable of explaining the data. As the result, BSDS shows greater robustness to the abrupt noisy and local changes in the data. Hence, BSDS typically requires fewer states to model the same data when compared to standard HMM based methods. These ,features help in the robust identification of brain states.

4 Performance of temporal clustering on n-back WM data

We conducted additional analysis using ICA in combination with temporal clustering, an approach widely used to investigate dynamic functional connectivity[25,26,27]. Briefly, this approach includes: (1) group-wise spatial ICA to identify components of interest, (2) extracting time series of components of interest, (3) applying a sliding window on time series and estimate time-varying covariance matrices, (4) clustering based on time-varying covariance matrix, and (5) determining the optimal number of clusters.

We used ICA maps from an independent study[19] because identifying the optimal number of components in spatial ICA and matching ICA components between two task sessions is non-trivial. Further, this approach also minimizes the impact of using individual spatial parcellations when comparing different methods and data across two sessions. The ICA maps here are the same as those used above to test the robustness of our findings with respect to ROI selection (**Supplementary Figure** 5). The ICA masks included bilateral AI, bilateral DLPFC, bilateral FEF, bilateral PPC, PCC, VMPFC and right DMPFC. We extracted time series from these regions and estimated dynamic functional interactions using a temporal sliding window approach with an exponentially decaying shape and a window length of 25 seconds (34 TRs) , which is shorter than the length of blocks (27.5 seconds), and a sliding step of 0.72 seconds (1 TR). Within each time window, we computed the z-transformed Pearson's correlation between the time-series taken pairwise. This resulted in a time-series of correlation matrices (T x C); here T is the number of time windows and C is number of pairwise interactions among regions at each time point. We then applied a group-wise k-mean clustering to the time-series of correlation matrices, with the number of clusters (k) ranging from 2 to 10. Twenty-five different initializations were used to reduce the chance of getting stuck in local minima. Clustering performance was estimated using the silhouette method and the optimal number of clusters was determined based on maximal silhouette across all the iterations[28].

Clustering analyses revealed that the optimal number of clusters was 2 in both data sessions (**Supplementary Figure** 16). Thus, despite the presence of three separate task conditions, only two dynamic brain states could be identified using this approach (**Supplementary Figure** 17). Further, we did not find any significant correlation between the occupancy rate of any latent brain states in the 2-back task blocks and WM accuracy ($p>0.6$, Pearson's correlation, **Supplementary Table** 17).

5 BSDS applied to HCP Relational Processing task

To demonstrate that BSDS can reliably estimate dynamic brain states in other cognitive domains, we applied BSDS to the HCP Relational Processing task. As described in detail below, we found that BSDS identifies brain states that are stable and replicable.

5.1 Data selection

The Human Connectome Project (HCP) Relational processing task fMRI data of 90 individuals were selected based on the following criteria: (1) range of head motion in any translational and rotational direction is less than 1 voxel; (2) average scan-to-scan head motion is less than 0.25 mm; (3) performance accuracy per session is greater than 50%; (4) criterion (1) – (3) must met in both sessions separately; and (5) subjects are right handed.

5.2 Relational Processing task

The Relational Processing task was adapted from a previous study[29]. In this task, there were two task conditions: a relational processing condition and a control matching condition, and stimuli with 6 different shapes and 6 different textures. In the relational processing condition, two pairs of stimuli were presented, with one pair at the top of the screen and the other pair at the bottom. Participants were told that they should first decide what dimension differs across the top pair of the stimuli (shape or texture) and then decide whether the stimuli at the bottom also differ along the same dimension. In the control matching condition, two stimuli were shown at the top of the screen and one at the bottom with a word in the middle of the screen (either shape or texture). Participants were told to decide whether the bottom stimulus matched either of the top two stimuli in that dimension. Each participant completed two runs of this task. Each run has three relational processing blocks, three control matching blocks and three 16-second fixation blocks. Each task block has 5 trials, lasting 18 seconds. Each stimulus was presented for 2800 ms, with a 400 ms ITI.

5.3 fMRI acquisition

For each individual, 232 frames were acquired in each session using multiband, gradient-echo planar imaging with the following parameters: RT, 720 ms; echo time, 33.1 ms; flip angle, $52°$; field of view, $280{\times}180$ mm; matrix, $140{\times}90$; and voxel dimensions, 2 mm isotropic.

5.4 fMRI preprocessing

Minimally preprocessed fMRI data for both sessions were obtained from the Human Connectome Project. Spatial smoothing with a Gaussian kernel of 6mm FWHM was first applied to the minimally preprocessed data to improve signal-to-noise ratio as well as anatomy correspondence between individuals. High-pass temporal filtering ($f{>}0.008$ Hz) was applied to remove low frequency signals related to scanner drift.

5.5 General linear model and contrast of interest

A conventional general linear model (GLM) analysis was conducted in order to determine relational-processing and matching related activation/deactivation peaks. Each block in each session was modeled as one of the two vectors: relational or match. The onset and duration of each vector were the onset and duration of the corresponding block. The contrast of interest was relational versus match.

5.6 Region of interest (ROI) and time series

ROIs were determined on the contrast of interest: relational versus match, including bilateral lateral occipital cortex (LOC), supramarginal gyrus (SMG), angular gyrus (AG), middle frontal gyrus (MFG), frontal pole (FP), medial frontal pole (mFP), right anterior insula (AI) and pre-supplementary motor area (preSMA) (**Supplementary Figure** 18). Each ROI was 6-mm radius sphere centered at the corresponding peak voxel. Time series of the 1st eigenvalue was extracted from each ROI. A multiple linear regression approach with 6 realignment parameters (3 translations and 3 rotations) was applied to time series to reduce head-motion-related artifacts and resulting time series was further linearly detrended and normalized.

5.7 Matching BSDS states between sessions

We applied BSDS to probe latent brain dynamics associated with ROIs in two data sessions separately. BSDS isolated five latent brain states in data Session 1 and four latent brain states in data Session 2. To determine whether one brain state identified in one session match one brain state identified in another session, we conducted cross-session brain state correlation analysis. Each brain state was defined by a covariate matrix in the latent space, estimated from the set of ROI activation timeseries in each session separately; and ROI timeseries can, in turn, be represented as a timeseries of posterior probabilities of estimated brain states. If a brain state in one session corresponds to a brain state in another session, then timeseries of posterior probabilities of these two brain states in the same data session should be highly correlated. Specifically, after obtaining four brain states in each session, we first computed posterior probability timeseries of each brain state in the data session from which brain states are estimated. An example is to compute posterior probability of State 1_1, estimated from Session 1 data. Next, we computed posterior probability timeseries of each brain state in the other data session. An example is to compute posterior probability of State 1_2, estimated from Session 2 data. Then, we computed correlation posterior probability timeseries of brain states, which were estimated from different sessions, in the same data session. For example, compute correlation between the posterior probability of State 1_1 in Session 1 and the posterior probability of State 1_2 in Session 1. High correlation would suggest that State 1_1 matches State 1_2 as the two states have highly similar posterior probability in the same data. Indeed, we found exclusive one-to-one mapping between four out of the five latent brain states from the data Session 1 and the four latent brain states from the data Session 2. The Pearson's correlation coefficients between the matched brain states across two sessions range from 0.65 to 0.94 (**Supplementary Table** 18). To simplify the report and improve the readability, we relabeled the matched four brain states in the two sessions to State 1, State 2, State 3 and State 4, and the fifth brain state in the data Session

1 to State 5.

5.8 Fractional occupancy of task-dominant latent brain states during task

We compared temporal evolution of latent brain states (**Supplementary Figure** 19a) and block structure of the experimental task (**Supplementary Figure** 19b). The latent brain states were only partially aligned with onset and offset of the three experimental task conditions (**Supplementary Figures** 19a, 19c). The relational, match and fixation conditions were each dominated by distinct brain states S4, S3 and S1 respectively (**Supplementary Figure** 19d). The brain state dominant (S4) during the relational task condition had an occupancy rate of $47.5{\pm}15.6\%$ in Session 1 and $50.9{\pm}15.6\%$ in Session 2. In both Sessions, the occurrence of the dominant state (S4) during the relational task condition was significantly higher than the occurrence of other non-dominant states (all p-values <0.001, two-tailed t-test). The mean lifetime of the dominant state (S4) in the relational condition was $7{\pm}2.6$ seconds in Session 1 and $7.4{\pm}2.7$ seconds in Session 2, significantly longer than the mean lifetime of the other non-dominant brain states (all p-values <0.001, two-tailed t-test), but much shorter than the 18 seconds task block (**Supplementary Figure** 19e). Thus, the relational condition is characterized by a mixture of brain states, with the dominant state active for only a relatively short interval. A similar pattern was observed for the states that were dominant in the match and fixation conditions (**Supplementary Figures** 19d, 19e). These results demonstrate that the relational processing task is characterized by latent task-induced states whose fractional occupancy is relatively short compared to the task blocks.

Supplementary Figures

Supplementary Figure 1: Directed acyclic graph representing the Bayesian switching dynamic systems (BSDS) model. $\boldsymbol{y}_t^s$ is the observed variable, $\boldsymbol{z}_t^s$ is the latent state variable, and $\boldsymbol{x}_{kt}^s$ is the latent source factor (latent space variable) associated to the k-th latent state variable at time t for subject s. (left) Allowed dependencies among observations, latent state variables and latent space variables. Given latent state and latent space variables, observations are conditionally independent. Latent states are connected to each other using a first-order Markov chain. Within a given state, latent space variables follow a dynamical process by an autoregressive model ($R{=}1$). (right) Generative model at time t and for subject s at a given latent state k, $z_{kt}^s{=}1$ (indicated with a red box). $\boldsymbol{\pi}$ and $\boldsymbol{A}$ are HMM parameters indicating the initial state probability distribution and the state transition probability distribution. $\boldsymbol{\mu}_k$ is the overall bias in the data, $\boldsymbol{\Psi}_k$ is the noise covariance matrix at state k, $\boldsymbol{u}_{kp}$ is the p-th column of the factor loading matrix and at the k-th state, and ν_{kp} is the prior on the variance of the $\boldsymbol{u}_{kp}$, known as ARD prior which controls the dimensionality of the latent subspace. $\vec{\boldsymbol{V}}_k$ indicates the autoregressive coefficients at the k-th state. $\boldsymbol{\Sigma}_k$ and $\boldsymbol{m}_k$ are the covariance and the mean in the latent space. Black boxes indicate replications.

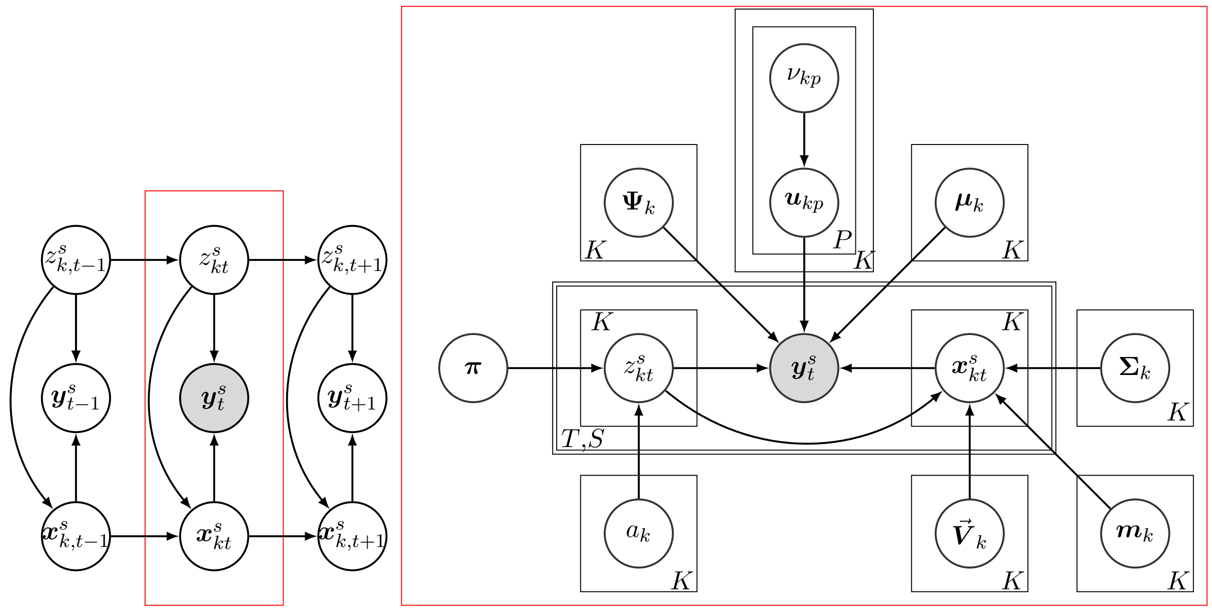

Supplementary Figure 2: Time-varying posterior probabilities of latent brain states predicts 2-back, 0-back and fixation task conditions. A linear SVM classifier was trained and performance of the classifier was tested within and between sessions. Permutation testing was used to evaluate statistical significance of cross-validation accuracies. We found significant prediction accuracy in **(a)** within-session cross-validation and **(b)** between-session cross-validation. $*$, $p{=}0.01$; $**$, $p{=}0.002$. Gray dash line indicates the chance level.

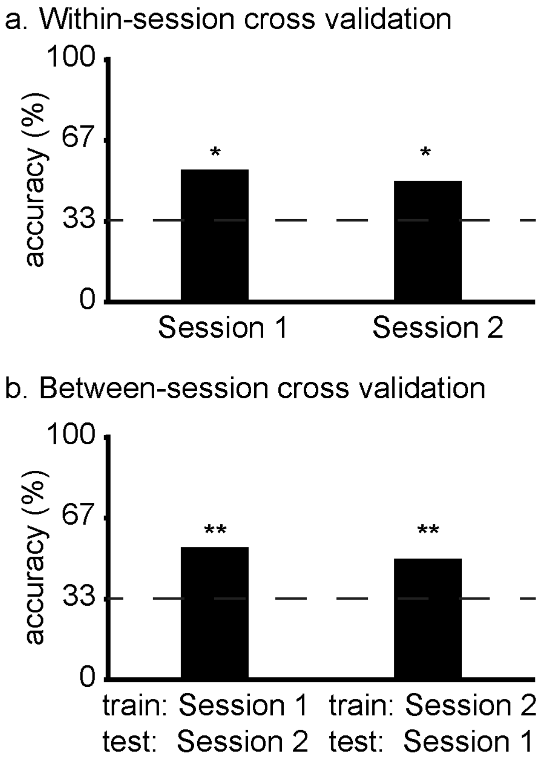

Supplementary Figure 3: Posterior probability of latent brain states in **(a)** 2-back and **(b)** 0-back task blocks. Time-varying posterior probabilities of each latent brain state were averaged across 2-back or 0-back task blocks in each participant. Each task block lasted about 28 seconds. Solid lines indicate averaged time-varying posterior probability across participants and shaded areas represent standard errors.

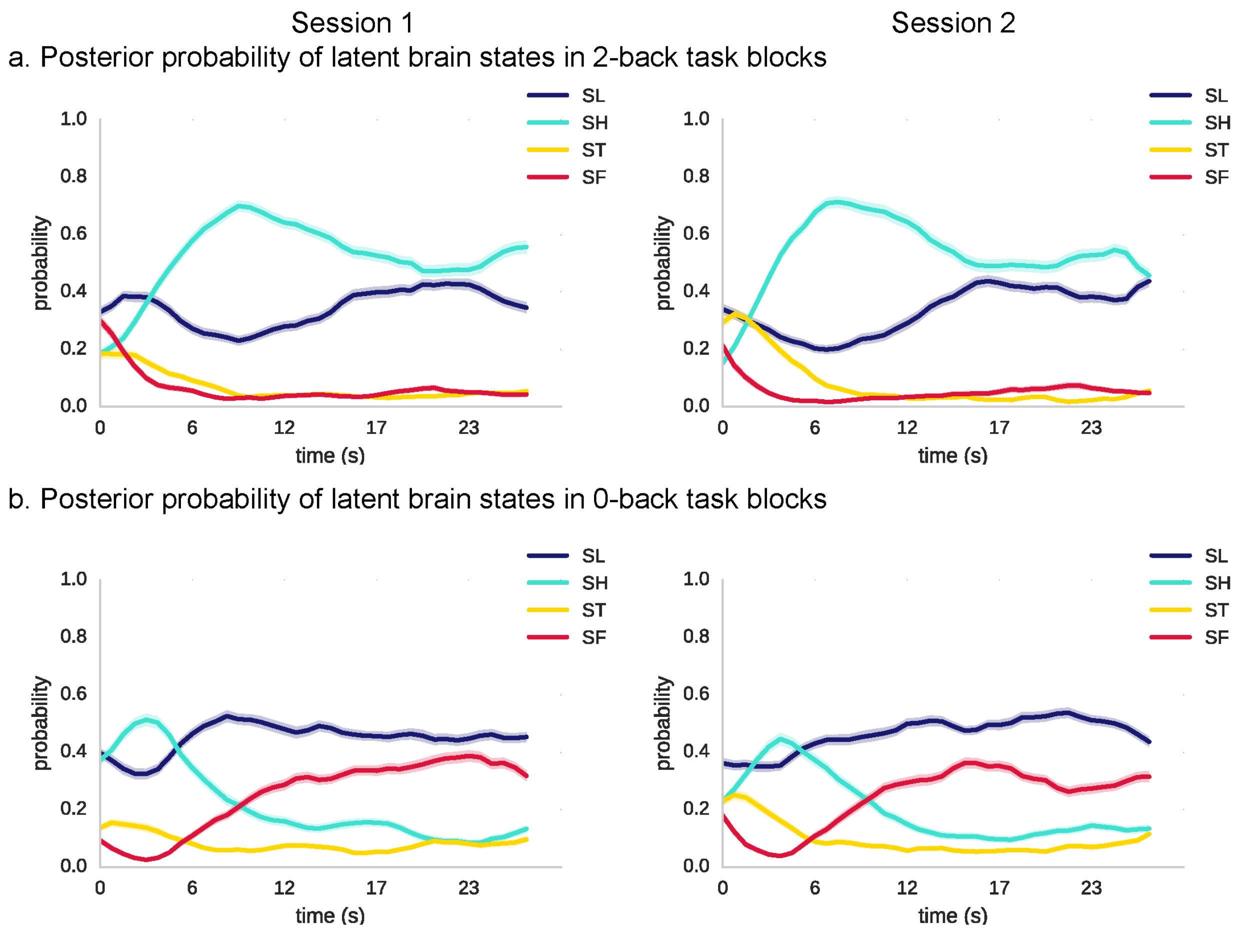

Supplementary Figure 4: Functional network measures of DMPFC in latent brain states. **(a)** DMPFC has higher node degree in ST than other latent brain states in both sessions. **(b)** DMPFC has higher betweenness centrality in ST than SL and SF in both sessions. $*$, $p<0.05$; $***$, $p<0.001$, two-tailed t-test. Error bar stands for standard deviation.

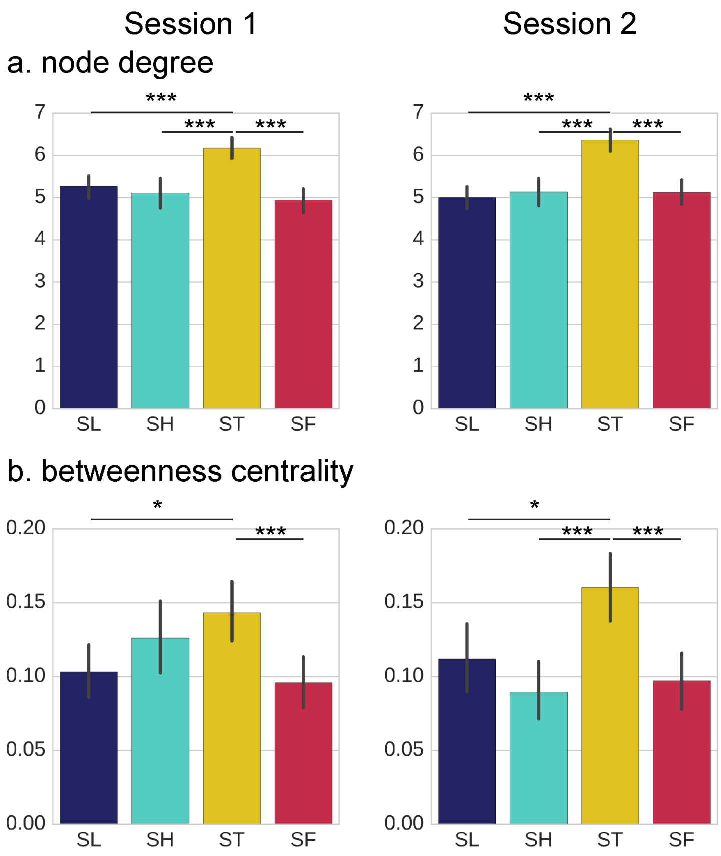

Supplementary Figure 5: BSDS applied to the n-back WM task data from the Human Connectome Project. Functional clusters from ICA components (Saliency network, Executive Control network and default mode network) were used based on[19]. ROIs in the analysis included bilateral anterior insula (AI), dorsolateral prefrontal cortex (DLPFC), frontal eye field (FEF), posterior parietal cortex (PPC), posterior cingulate cortex (PCC), ventromedial prefrontal cortex (VMPFC) and dorsomedial prefrontal cortex (DMPFC).

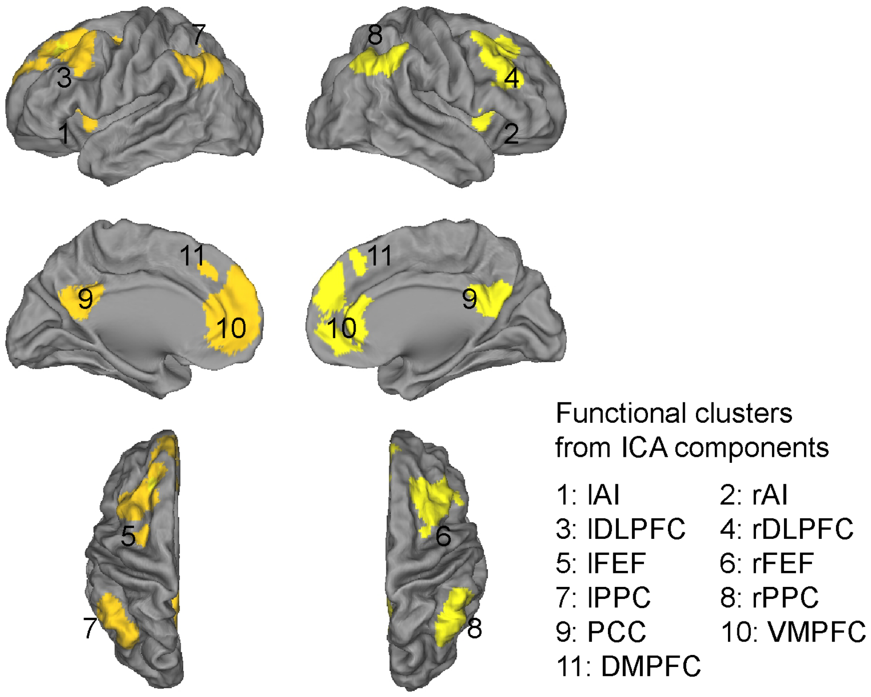

Supplementary Figure 6: BSDS applied on data of ROIs shown in Figure S5 revealed latent brain states during WM, their dynamic properties and replication across Sessions 1 and 2. **(a)** Temporal evolution of the four latent brain states identified in each of the 122 participants. **(b)** Corresponding task waveforms of the three task conditions in the n-back WM task – 0-back, 2-back and fixation blocks – are shown in the same layout. **(c)** Time-varying posterior probability of each latent brain state across participants. **(d)** Occupancy rates of latent brain states for the three states which dominate the 0-back, 2-back, and fixation task blocks, SL, SH and SF respectively. **(e)** Mean lifetimes of latent brain states for the three states which dominate the 0-back, 2-back and fixation task blocks, SL, SH and SF respectively (p-values <0.001, two-tailed t-test). **(f)** BSDS revealed a novel transition state ST, which occurs more frequently right after the onset of experimental task blocks than other latent states in both sessions (p-values <0.05, two-tailed t-test) but not to SG in Session 1. Note that SL, SH, ST, SF and SG were named by their task dominancy in panel **c-f**, which correspond to S1, S2, S3, S4 and S5 in panel **a**. Color code mapping in panels **a**, **c**, **d**, **e** and **f**: dark blue – SL, cyan – SH, yellow – ST, red – SF, purple – SG. Color code mapping in panel **b**: dark blue – 0-back, cyan – 2-back, red – fixation.

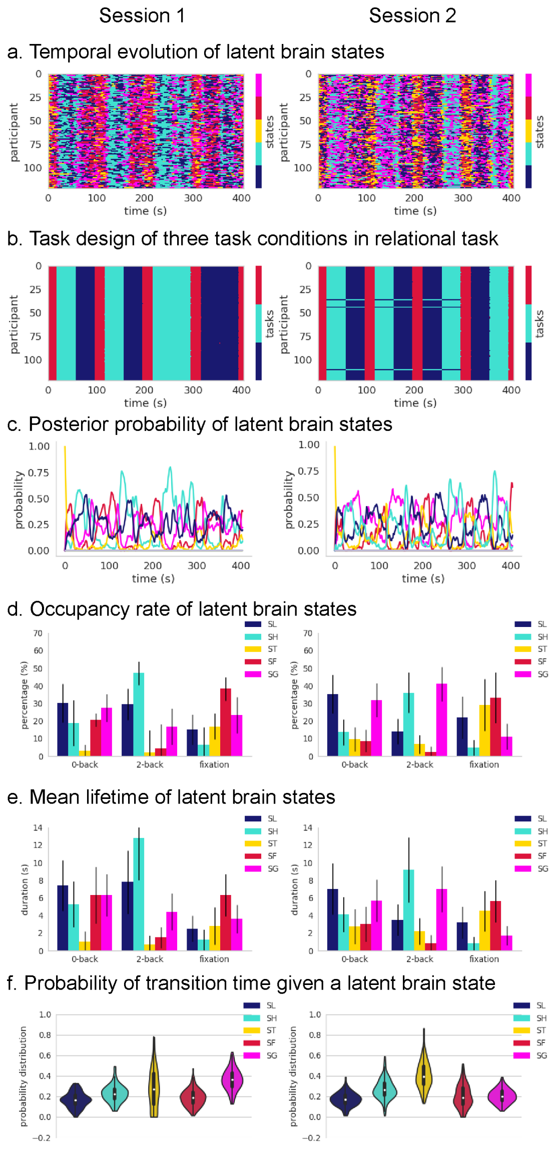

Supplementary Figure 7: Occupancy rate and mean lifetimes of latent brain states from BSDS applied on data from ROIs shown in **Supplementary Figure** 5 predict WM performance: replication across Sessions 1 and 2. **(a)** A multiple linear regression model was trained using occupancy rates of latent brain states in the 2-back task to predict WM accuracy. A significant association was observed and predicted accuracies were correlated with observed accuracy in both sessions: (Session 1: $r\!=\!0.29$, $p\!<\!0.002$; Session 2: $r\!=\!0.26$, $p\!<\!0.005$, Pearson's correlation). **(b)** Occupancy rate of the latent brain state SH which dominates the 2-back WM task condition was correlated with WM task accuracy in both sessions (Session 1: $r\!=\!0.31$, $p\!<\!0.001$; Session 2: $r\!=\!0.25$, $p\!<\!0.005$, Pearson's correlation). No such relations were found for any of the other latent states. **(c)** A multiple linear regression model was trained using mean lifetimes of latent brain states in the 2-back task to predict WM accuracy. Here again, a significant association was found and the predicted accuracy was correlated with observed accuracy in both sessions (Session 1: $r\!=\!0.22$, $p\!<\!0.05$; Session 2: $r\!=\!0.24$, $p\!<\!0.01$, Pearson's correlation). **(d)** Mean lifetime of the latent brain state SH which dominates the 2-back WM task condition was correlated with WM task accuracy in Session 2 ($r\!=\!0.20$, $p\!<\!0.05$, Pearson's correlation) but marginally in Session 2 ($p\!=\!0.06$, Pearson's correlation). Shaded area represents 95% confidence interval. Color code mapping in panels **b** and **d**: dark blue – SL, cyan – SH, yellow – ST, red – SF, purple – SG.

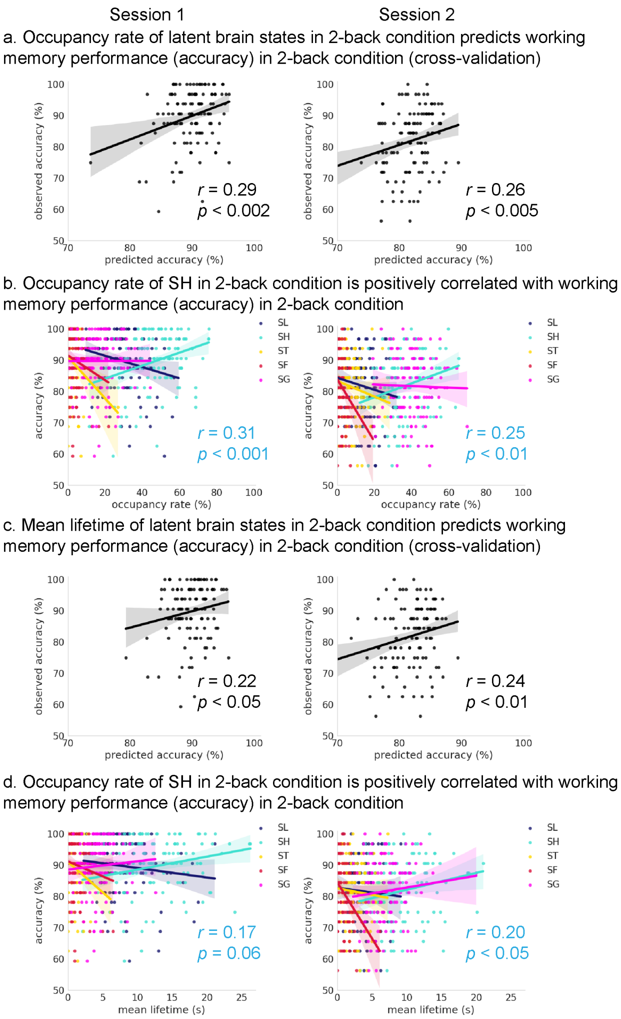

Supplementary Figure 8: BSDS applied on data using 12 motion parameters regression revealed latent brain states during WM, their dynamic properties and replication across Sessions 1 and 2. **(a)** Temporal evolution of the four latent brain states identified in each of the 122 participants. **(b)** Corresponding task waveforms of the three task conditions in the n-back WM task – 0-back, 2-back and fixation blocks – are shown in the same layout. **(c)** Time-varying posterior probability of each latent brain state across participants. **(d)** Occupancy rates of latent brain states for the three states which dominate the 0-back, 2-back, and fixation task blocks, SL, SH and SF respectively. **(e)** Mean lifetimes of latent brain states for the three states which dominate the 0-back, 2-back and fixation task blocks, SL, SH and SF respectively (p-values <0.001, Pearson's correlation). **(f)** BSDS revealed a novel transition state ST, which occurs more frequently right after the onset of experimental task blocks than other latent states in both sessions (p-values <0.05, Pearson's correlation) except that it is marginally significant compared to SH in Session 1 ($p=0.07$, Pearson's correlation). Note that SL, SH, ST, SF and SG were named by their task dominancy in panel **c-f**, which correspond to S1, S2, S3, S4 and S5 in panel **a**. Color code mapping in panels **a**, **c**, **d**, **e** and **f**: dark blue – SL, cyan – SH, yellow – ST, red – SF, purple – SG. Color code mapping in panel **b**: dark blue – 0-back, cyan – 2-back, red – fixation.

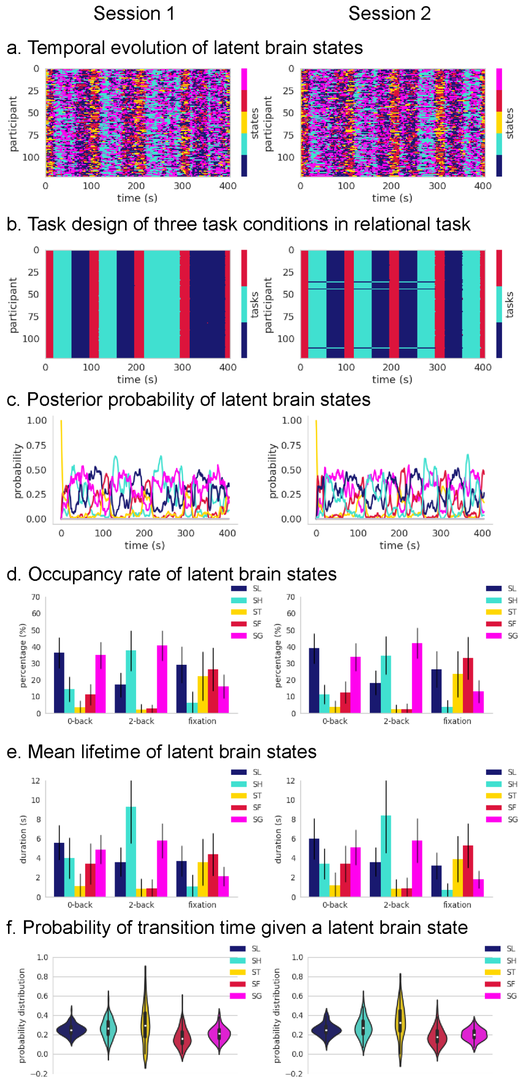

Supplementary Figure 9: Occupancy rate and mean lifetimes of latent brain states from BSDS with 12 motion regression predict WM performance: replication across Sessions 1 and 2. **(a)** A multiple linear regression model was trained using occupancy rates of latent brain states in the 2-back task to predict WM accuracy. A significant association was observed and predicted accuracies were correlated with observed accuracy in both sessions: (Session 1: $r{=}0.29$, $p{<}0.002$; Session 2: $r{=}0.31$, $p{<}0.001$, Pearson's correlation). **(b)** Occupancy rate of the latent brain state SH which dominates the 2-back WM task condition was correlated with WM task accuracy in both sessions (Session 1: $r{=}0.33$, $p{<}0.001$; Session 2: $r{=}0.27$, $p{<}0.005$, Pearson's correlation). No such relations were found for any of the other latent states. **(c)** A multiple linear regression model was trained using mean lifetimes of latent brain states in the 2-back task to predict WM accuracy. Here again, a significant association was found and the predicted accuracy was correlated with observed accuracy in both sessions (Session 1: $r{=}0.25$, $p{<}0.01$; Session 2: $r{=}0.24$, $p{<}0.01$, Pearson's correlation). **(d)** Mean lifetime of the latent brain state SH which dominates the 2-back WM task condition was correlated with WM task accuracy in Session 1 (Session 1: $r{=}0.23$, $p{=}0.01$, Pearson's correlation) but not in Session 2. Shaded area represents 95% confidence interval. Color code mapping in **b** and **d**: dark blue – SL, cyan – SH, yellow – ST, red – SF, purple – SG.

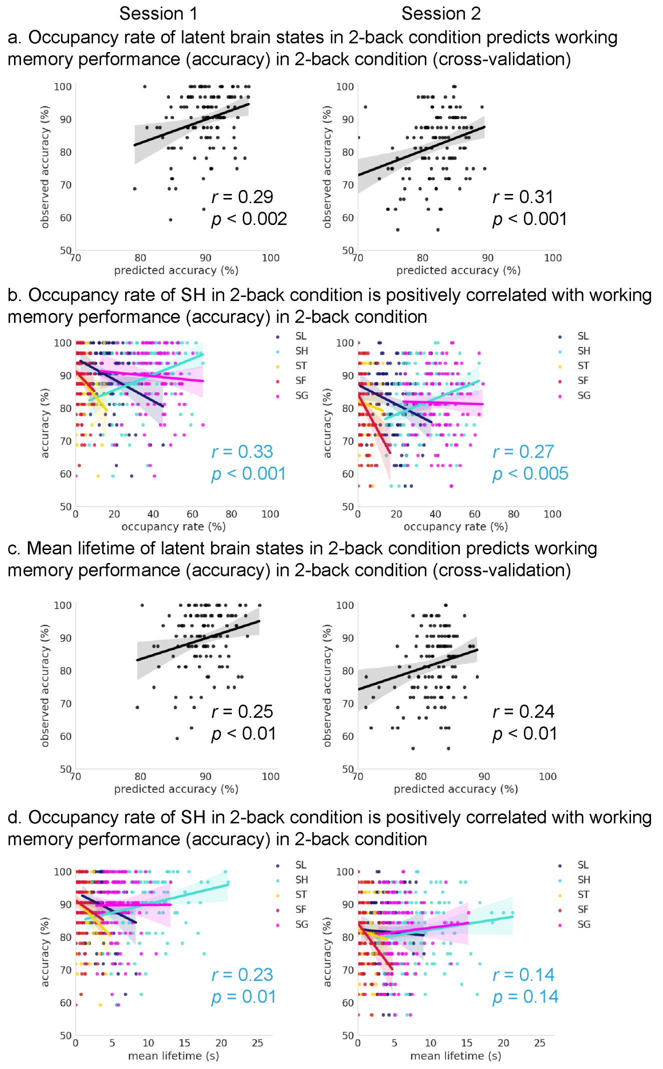

Supplementary Figure 10: HDP-HMM.A on opto-fMRI dataset. (top) ROI timeseries from the first subject, (middle) stimulation design which is the same for all three subjects, (bottom) estimated temporal evolution of states for each subject. HDP-HMM.A converged to three states.

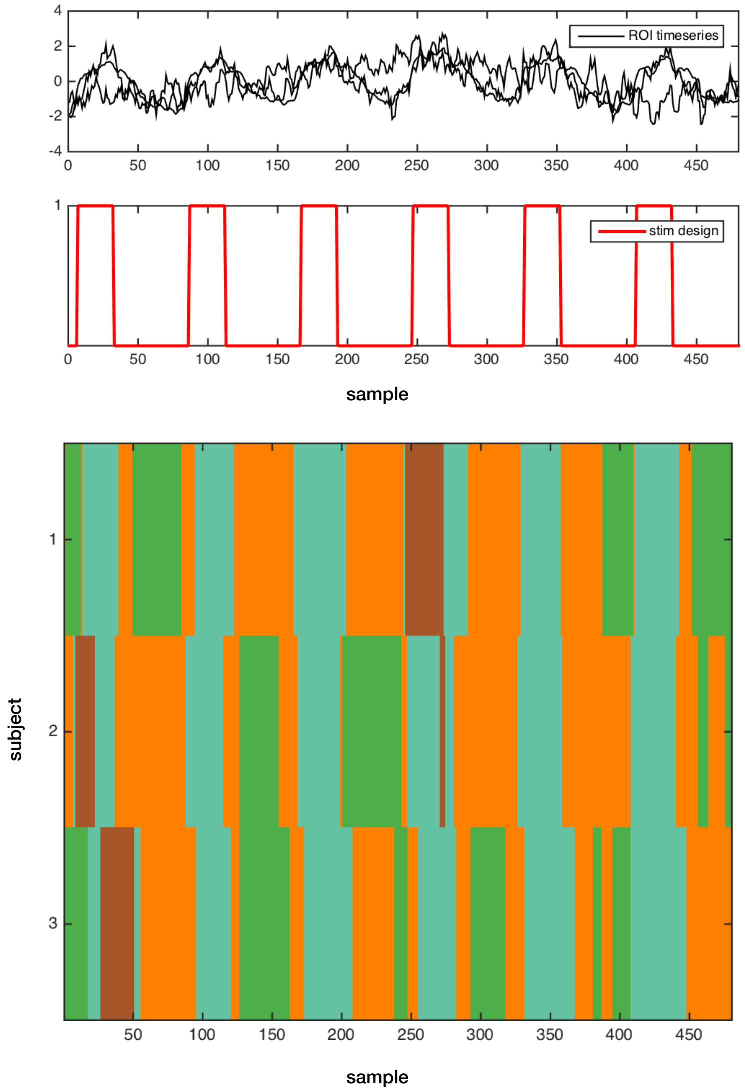

Supplementary Figure 11: HDP-HMM.B on opto-fMRI dataset. (top) timeseries, (middle) stimulation design which is the same for all three subjects, (bottom) estimated temporal evolution of states for each subject. HDP-HMM.B converged to three states.

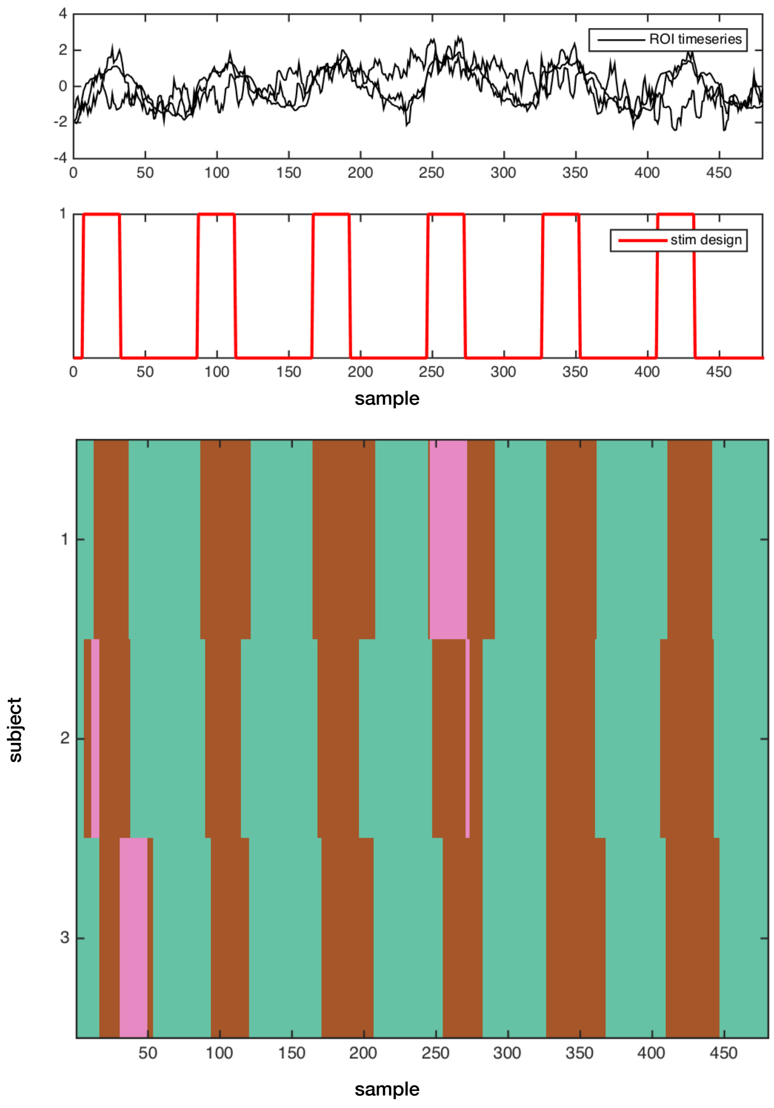

Supplementary Figure 12: BSFA on opto-fMRI dataset. (top) ROI timeseries from the first subject, (middle) stimulation design which is the same for all three subjects, (bottom) estimated temporal evolution of states for each subject. BSFA was initialized with 20 states and it converged to 14 states.

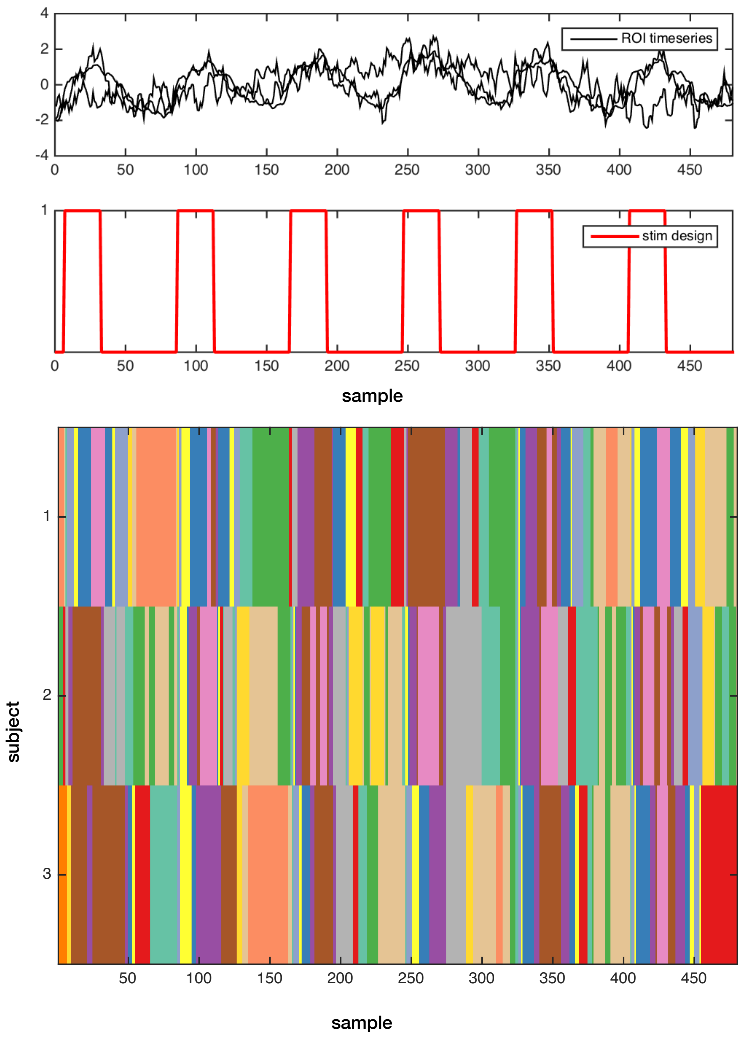

Supplementary Figure 13: HDP-HMM.A on WM dataset. (top) ROI timeseries from the first subject, (middle) stimulation design for all subjects, (bottom) estimated temporal evolution of states for each subject. HDP-HMM.A converged to 10 states in Session 1 and to 9 states in Session 2. Note that there is no one-to-one correspondence between color codes across sessions and task designs.

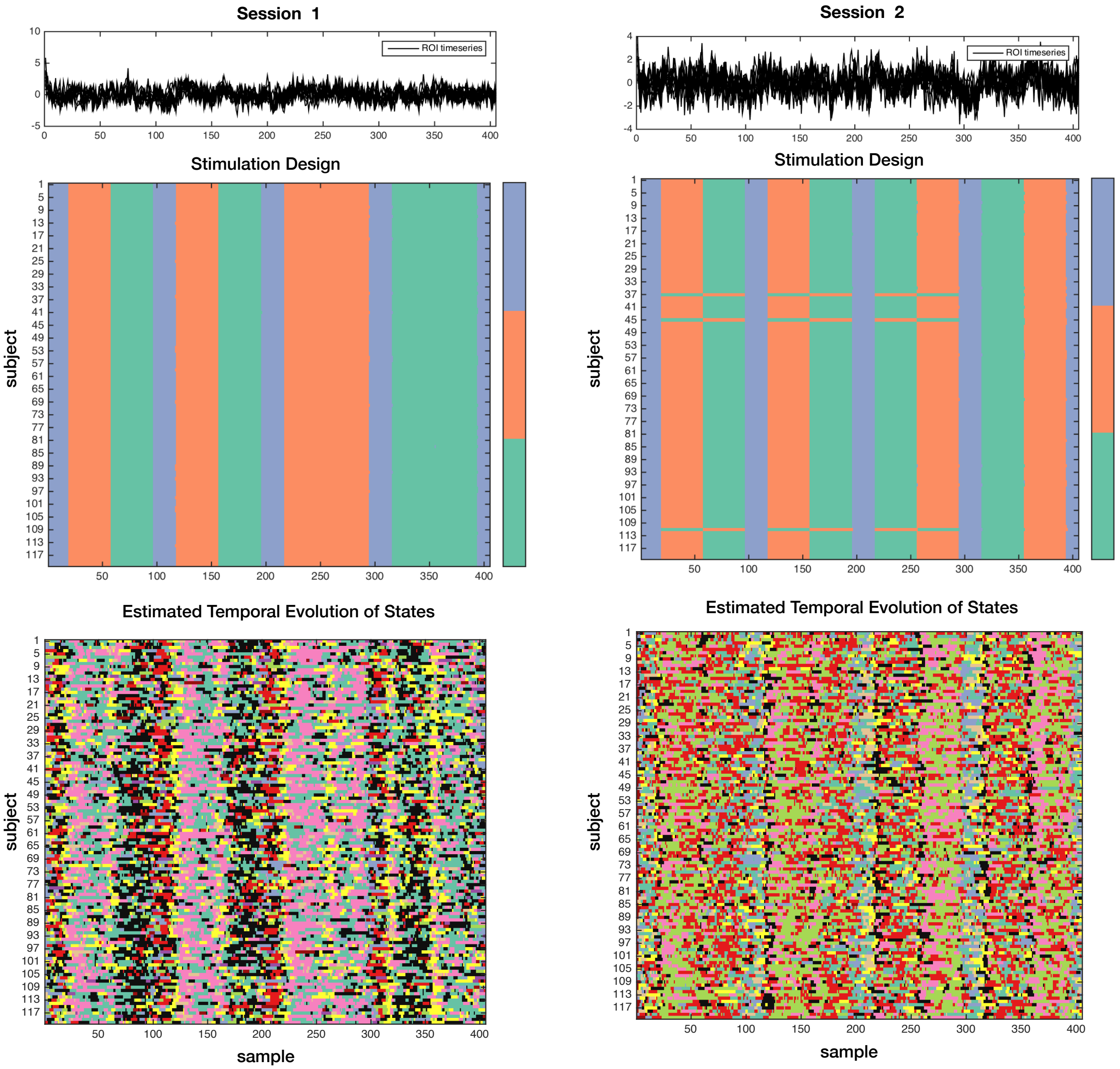

Supplementary Figure 14: HDP-HMM.B on WM dataset. (top) ROI timeseries from the first subject, (middle) stimulation design for all subjects, (bottom) estimated temporal evolution of states for each subject. HDP-HMM.B converged to 10 states in Session 1 and to 10 states in Session 2. Note that there is no one-to-one correspondence between color codes across sessions and task designs.

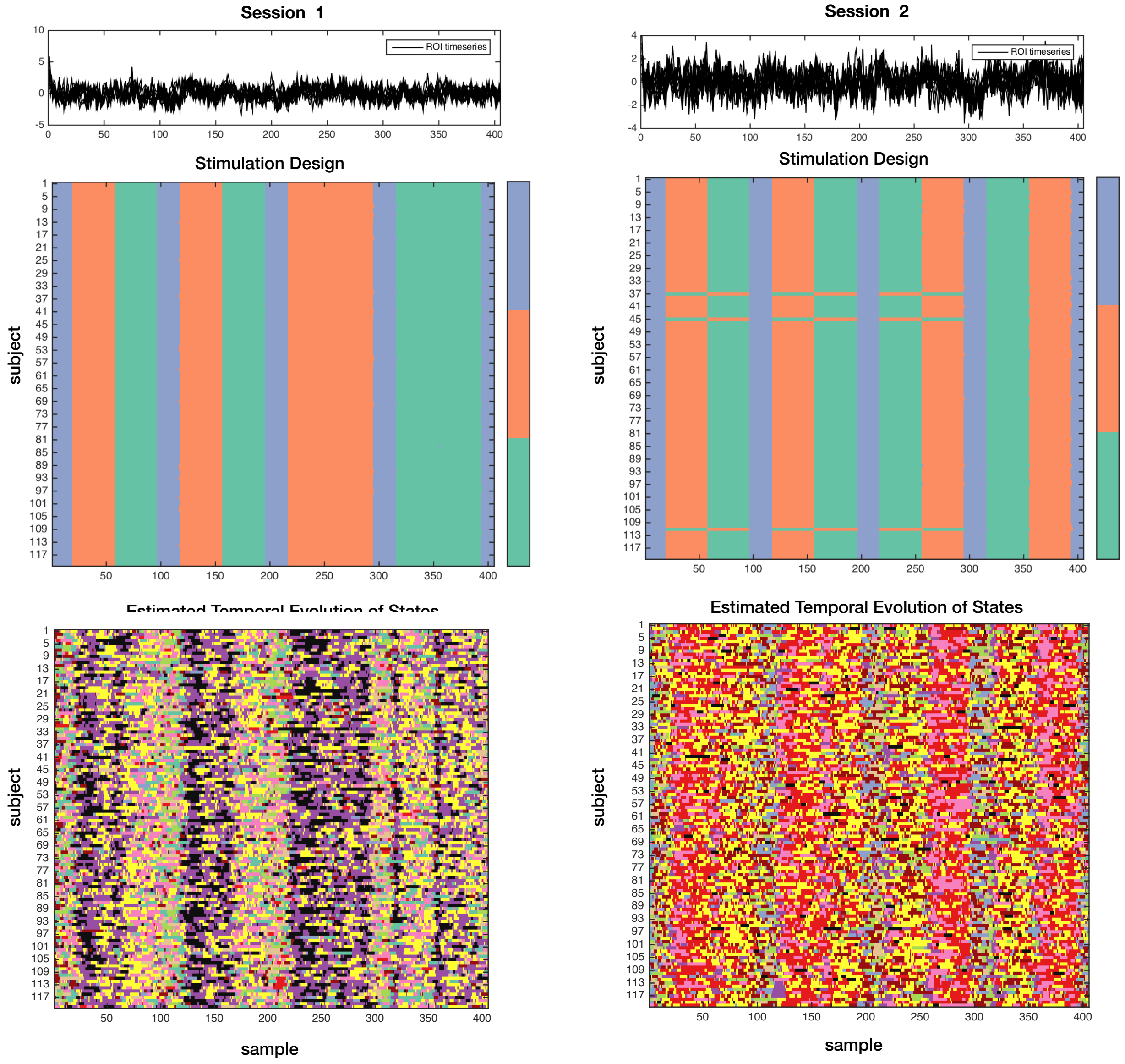

Supplementary Figure 15: BSFA on WM dataset. (top) ROI timeseries from the first subject, (middle) stimulation design for all subjects, (bottom) estimated temporal evolution of states for each subject. BSFA converged to 15 states in Session 1 and to 16 states in Session 2. Note that there is no one-to-one correspondence between color codes across sessions and task designs.

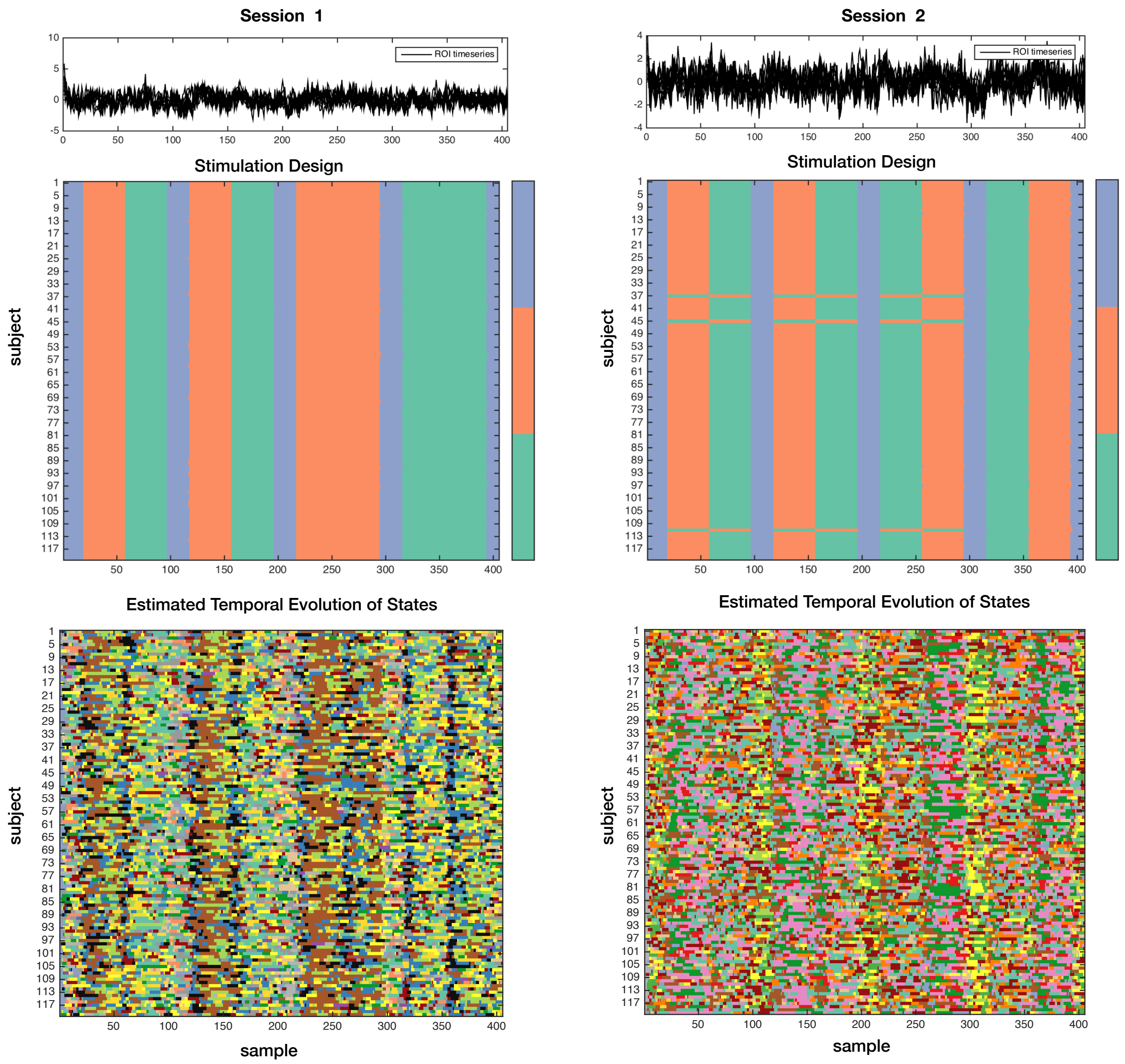

Supplementary Figure 16: The optimal number of temporal clusters was determined using the maximal silhouette obtained across multiple iterations. In both data sessions (n-back task), the maximal silhouette value was 2. Silhouette is a measure for validating clustering, which evaluates how similar a data point is to its own cluster compared to other clusters. Each color represents a k-mean clustering performance with a random initialization (the number of clusters ranges from 2 to 10).

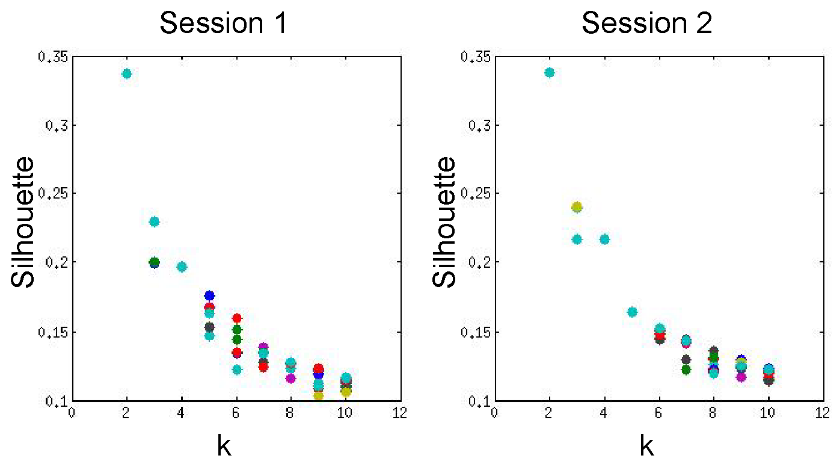

Supplementary Figure 17: Two temporal states were identified in each data session using the temporal clustering approach (HCP n-back task).

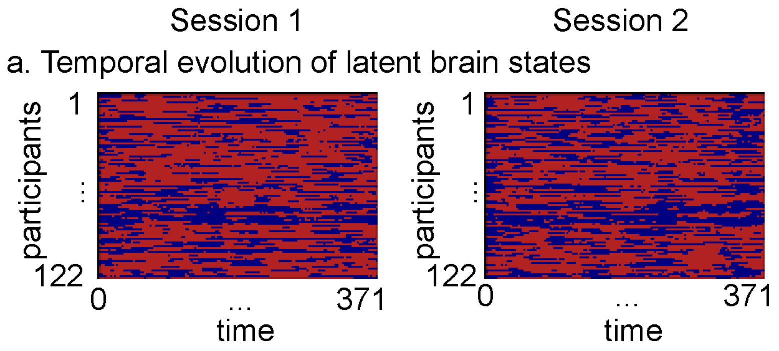

Supplementary Figure 18: BSDS applied to the Relational Processing task data from the Human Connectome Project. **(a)** Brain regions activated (warm colors) and deactivated (cool colors) during the relational processing, compared to the control matching, condition. **(b)** Regions of interest (ROIs) were determined using activation and deactivation peaks from (a). ROIs activated during relational processing and control matching tasks: 1, left lateral occipital cortex (lLOC); 2, right lateral occipital cortex (lLOC); 3, left supramarginal gyrus (lSMG); 4, right supramarginal gyrus (rSMG); 5, left angular gyrus (lAG); 6, right angular gyrus (rAG); 7, left middle frontal gyrus (lMFG); 8 right middle frontal gyrus (rMFG); 9, left frontal pole (lFP); 10, right frontal pole (rFP); 11, medial frontal pole (mFP); 12 right anterior insula (rAI); and 13, pre-supplementary motor area (preSMA).

a. Task effect (relation vs. match)

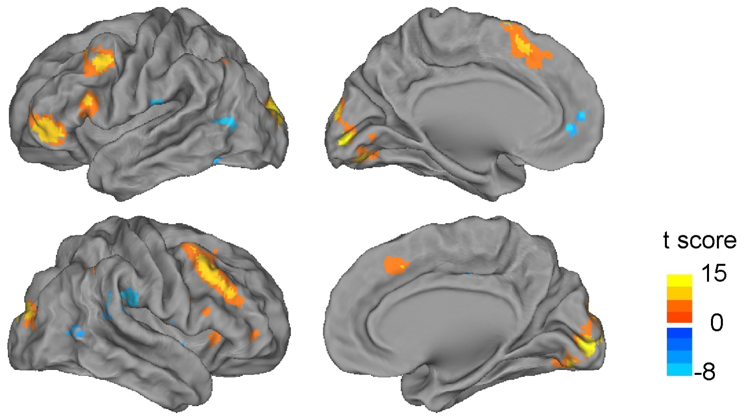

b. Regions of interest

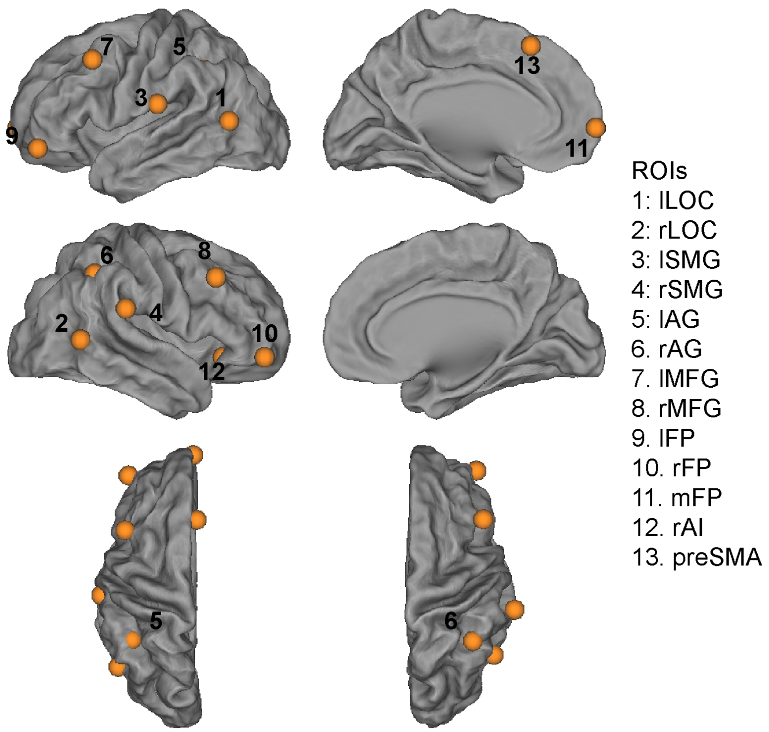

Supplementary Figure 19: Latent brain states during a Relational Processing task, their dynamic properties and replication across Sessions 1 and 2. **(a)** Temporal evolution of the latent brain states identified in each of the 90 participants. **(b)** Corresponding task waveforms of the three task conditions in the relational processing task – relational, match and fixation blocks – are shown in the same layout. **(c)** Time-varying posterior probability of each latent brain state across participants. **(d)** Occupancy rates of latent brain states for the three states which dominate the relational, match, and fixation task blocks, S4, S3 and S1 respectively. **(e)** Mean lifetimes of latent brain states for the three states which dominate the relational, match and fixation task blocks, S4, S3 and S1 respectively (p-values <0.001, two-tailed t-test). **(f)** Probability of transition time given a latent brain state. Color code mapping in panels **a**, **c**, **d**, **e** and **f**: dark blue – S1, cyan – S2, yellow – S3, red – S4, purple – S5. Color code mapping in panel **b**: dark blue – control match, red – relational processing, cyan – fixation.

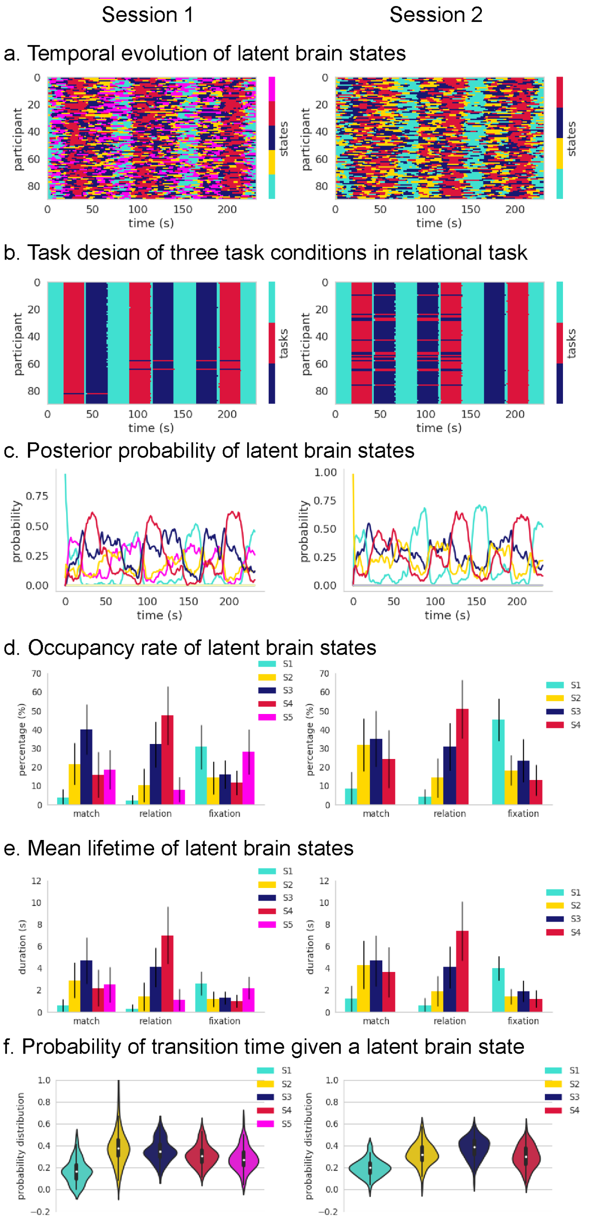

Supplementary Tables

Supplementary Table 1: Table of Notations.

		Observed Variables
S	$\triangleq$	number of subjects, $s{=}1,\ldots,S$
T	$\triangleq$	number of measurements, $t{=}1,\ldots,T$
D	$\triangleq$	number of regions of interest (ROIs), $d{=}1,\ldots,D$
y^s_{td}	$\triangleq$	scalar observed variable at the d-th ROI in time t for subject s
$\boldsymbol{y}^s_t{=}(y^s_{t1},\ldots,y^s_{1D})^\top$	$\triangleq$	D-dimensional observation vector at time t, subject s
$\boldsymbol{Y}^s{=}\{\boldsymbol{y}^s_1,\ldots,\boldsymbol{y}^s_T\}$	$\triangleq$	sequence of T measurements
$\underline{\boldsymbol{Y}}{=}\{\boldsymbol{Y}^s\vert s{=}1,\ldots,S\}$	$\triangleq$	sequence of T measurements from S subjects
		Latent State Variables
K	$\triangleq$	number of latent state variables, $k{=}1,\ldots,K$
z^s_{kt}	$\triangleq$	the k-th latent state variable in time t for subject s
$\boldsymbol{z}^s_t{=}(z^s_{1t},\ldots,z^s_{Kt})^\top$	$\triangleq$	1–of–K discrete vector of latent state variables in time t for subject s
$\boldsymbol{Z}^s{=}\{\boldsymbol{z}^s_1,\ldots,\boldsymbol{z}^s_T\}$	$\triangleq$	sequence of T latent state variables
$\underline{\boldsymbol{Z}}{=}\{\boldsymbol{Z}^s\vert s{=}1,\ldots,S\}$	$\triangleq$	sequence of T latent state variables from S subjects
		Latent Space Variables
P	$\triangleq$	number of latent space variables, $p{=}1,\ldots,P$
x^s_{pkt}	$\triangleq$	the p-th latent space variable in time t for subject s at the k-th latent state
$\boldsymbol{x}^s_{kt}{=}(x^s_{1kt},\ldots,x^s_{Pkt})^\top$	$\triangleq$	P-dimensional vector of latent space variables in time t for subject s
$\boldsymbol{X}^s_k{=}\{\boldsymbol{x}^s_{k1},\ldots,\boldsymbol{x}^s_{kT}\}$	$\triangleq$	sequence of T latent space variables
$\underline{\boldsymbol{X}}{=}\{\boldsymbol{X}^s\vert s{=}1,\ldots,S\}$	$\triangleq$	sequence of T latent state variables from S subjects
		HMM Variables
$\boldsymbol{\theta}^{\text{HMM}}{=}\{\boldsymbol{\pi},\boldsymbol{A}\}$	$\triangleq$	set of HMM parameters
$\boldsymbol{\pi}$	$\triangleq$	initial state probability distribution
$\boldsymbol{A}$	$\triangleq$	state transition probability distribution
$\boldsymbol{O}^s_t$	$\triangleq$	emission probability distribution in time t for subject s
		Factor Analysis Variables
$\boldsymbol{\theta}^{\text{FA}}{=}\{\boldsymbol{\mu},\boldsymbol{U},\boldsymbol{\Psi}\}$	$\triangleq$	set of factor analysis parameters
$\boldsymbol{\mu}$	$\triangleq$	overall bias vector
$\boldsymbol{U}$	$\triangleq$	linear transformation matrix
$\boldsymbol{\Psi}$	$\triangleq$	noise covariance matrix
		Autoregressive Process Variables
$\boldsymbol{\theta}^{\text{AR}}{=}\{\vec{\boldsymbol{V}},\boldsymbol{\Sigma},\boldsymbol{m}\}$	$\triangleq$	set of autoregressive process parameters
$\vec{\boldsymbol{V}}$	$\triangleq$	autoregressive coefficients vector
$\boldsymbol{\Sigma}$	$\triangleq$	noise covariance matrix
$\boldsymbol{m}$	$\triangleq$	noise mean vector

Supplementary Table 2: Algorithmic summary of BSDS using noninformative initialization.

Algorithm 1 Bayesian Switching Dynamical Systems

step 0

Set the number of states, K (the initial value of K is usually set to a large value, and during learning those states with small contributions will get weights close to zero);

Set the intrinsic dimensionality of the latent subspace, P, (in general P is set to be smaller than data dimension, $P<D$, however, in a fully noninformative initialization, one can simply set $p=D-1$).

step 1: initialization

- set the prior distribution parameters using § 1.2;
- initialize $\langle \boldsymbol{z}_t^s \rangle_{q(\boldsymbol{z}_t^s)}$ (e.g., using K-means algorithm, with Euclidean distance as the similarity measure, initialized using K clusters).

repeat

step 2: optimization of the model parameters

- optimize $q(\boldsymbol{\theta}_k^{\mathsf{FA}}), \forall k=1,\ldots,K$ using § 1.4.2;
- optimize $q(\boldsymbol{\theta}_k^{\mathsf{AR}}), \forall k=1,\ldots,K$ using § 1.4.3.
- optimize $q(\boldsymbol{\theta}_k^{\mathsf{HMM}}), \forall k=1,\ldots,K$ using § 1.4.1.

step 3: optimization sufficient statistics of the latent variables

- optimize sufficient statistics of the latent space variables using § 1.6.1;
- optimize sufficient statistics of the latent state variables using § 1.6.2.

step 4: optimization of the posterior hyperparameters

- update posterior hyperparameters using § 1.5.

step 5: check for convergence

- evaluate the convergence by monitoring the lower bound value at each iteration given by Eq. (8) and terminate the optimization if the change in the lower bound values in the current iteration and the previous iteration is smaller than a small threshold (e.g., 10^{-3}) or we have reached the maximum number of allowed iterations.

until convergence

Supplementary Table 3: Subject-level learning using BSDS.

Algorithm 2 Subject-level analysis using informative priors computed from group-level analysis

---

step 0
Set the number of states, K, and the intrinsic dimensionality of the latent subspace, P, from the given group-model computed using Algorithm 1.
step 1: initialization

- set the prior distribution parameters using $\S$ 1.2;
- initialize $\langle \boldsymbol{z}_t^s \rangle_{q(\boldsymbol{z}_t^s)}$ using $\langle z_{kt}^s \rangle_{q(z_{kt}^s)}$,$\forall k$, from the group-level analysis.

repeat
step 2: optimization of the model parameters

- optimize $\{q(\boldsymbol{\theta}_k^{\mathsf{FA}})\}^s$,$\forall k$=1,...,$K$ using $\S$ 1.4.2;
- optimize $\{q(\boldsymbol{\theta}_k^{\mathsf{AR}})\}^s$,$\forall k$=1,...,$K$ using $\S$ 1.4.3.
- optimize $\{q(\boldsymbol{\theta}_k^{\mathsf{HMM}})\}^s$,$\forall k$=1,...,$K$ using $\S$ 1.4.1.

step 3: optimization sufficient statistics of the latent variables

- optimize sufficient statistics of the latent space variables using $\S$ 1.6.1;

step 4: optimization of the posterior hyperparameters

- update posterior hyperparameters using $\S$ 1.5.

step 5: check for convergence

- evaluate the convergence by monitoring the lower bound value at each iteration given by Eq. (8) and terminate the optimization if the change in the lower bound values in the current iteration and the previous iteration is smaller than a small threshold (e.g., 10^{-3}) or we have reached the maximum number of allowed iterations.

until convergence

Supplementary Table 4: Correlation of posterior probabilities of latent brain states from training and test sessions.

		**Session 2 test state**			
		3	7	8	9
**Session 1**	1	**0.96**	-0.17	-0.36	-0.49
**training state**	2	-0.45	-0.21	-0.44	**0.98**
	3	0.25	**0.83**	-0.11	-0.17
	8	0.38	0.16	**0.96**	-0.43

		**Session 1 test state**			
		1	2	3	8
**Session 2**	3	**0.96**	-0.44	-0.24	-0.37
**training state**	7	-0.17	-0.23	**0.84**	-0.15
	8	-0.36	-0.45	-0.14	**0.96**
	9	-0.48	**0.98**	-0.17	-0.44

**Matched latent state**	**Session 1**	**Session 2**
1	1	3
2	2	9
3	3	7
4	8	8

Supplementary Table 5: Occupancy rate (%) of latent brain states.

**Latent brain state**	**Session 1** mean(std)	**Session 2** mean(std)
1	37(6)	37(6)
2	30(6)	30(5)
3	10(5)	10(4)
4	23(4)	23(4)

Supplementary Table 6: Counts of state switching paths between states SH and SF in each condition across pooled participants and blocks.

Path	Session 1			Session 2		
	0-back	2-back	fixation	0-back	2-back	fixation
SH to SF						
SH-SL-SF	231	64	56	233	82	87
SH-ST-SF	28	11	37	20	6	36
SH-SL-ST-SF	15	5	4	7	2	1
SH-ST-SL-SF	15	2	3	12	2	2
SH-SL-ST-SL-SF	7	1			3	
SH-ST-SL-ST-SF	1		1	1		1
SH-SL-ST-SL-ST-SF	2			1		
SH-ST-SL-ST-SL-SF	1					
SF to SH						
SF-SL-SH	94	171	35	106	110	42
SF-ST-SH	9	24	7	13	29	11
SF-SL-ST-SH	2	3	2	2	2	2
SF-ST-SL-SH	3	7	1	8	6	
SF-SL-ST-SL-SH		3	1	1	1	
SF-ST-SL-ST-SH					1	

Supplementary Table 7: Cross-validation accuracy and significance for prediction of brain states using logistic regression classifier trained on connectivity features common between Sessions 1 and 2.

States classified	Session 1		Session 2	
	accuracy	p-value	accuracy	p-value
SL vs SH	74.20%	0.002	77.90%	0.002
SL vs ST	96.70%	0.002	97.50%	0.002
SL vs SF	91.40%	0.002	90.60%	0.002
SH vs ST	95.90%	0.002	93.90%	0.002
SH vs SF	84.00%	0.002	80.00%	0.002
ST vs SF	88.90%	0.002	91.00%	0.002

Supplementary Table 8: Correlation between occupancy rates of latent brain states in the 2-back task and WM accuracy.

Latent state	Session 1		Session 2	
	r	*p*	*r*	*p*
SL	-0.32	0.001	-0.16	0.07
SH	0.39	0.001	0.31	0.001
ST	-0.18	0.04	-0.11	0.22
SF	-0.19	0.03	-0.37	0.001

Supplementary Table 9: Correlation between mean lifetimes of latent brain states in the 2-back task and WM accuracy.

Latent state	Session 1		Session 2	
	r	*p*	*r*	*p*
SL	-0.2	0.03	-0.15	0.11
SH	0.37	0.001	0.27	0.003
ST	-0.15	0.1	-0.07	0.47
SF	-0.15	0.11	-0.37	0.001

Supplementary Table 10: Multiple linear regression revealed that the posterior probability of the state SH was the most robust predictor of WM performance in the 2-back task.

	Beta	***t value***	***p value***
**Session 1**			
posterior probability of state SH in 2-back	0.24	4.31	< 0.001
Age	0.002	0.95	0.35
Gender	0.03	1.8	0.07
Ethnicity	-0.02	-1.06	0.29
**Session 2**			
posterior probability of state SH in 2-back	0.27	4.05	< 0.001
Age	-0.001	-0.7	0.48
Gender	0.04	2.3	0.02
Ethnicity	-0.03	-1.25	0.2

Supplementary Table 11: Spearman correlation between occupancy rates of latent brain states in the 2-back task and WM accuracy.

**Latent state**	**Session 1**		**Session 2**	
	r	*p*	*r*	*p*
SL	-0.27	0.003	-0.13	0.13
SH	0.32	0.001	0.29	0.001
ST	-0.19	0.04	-0.12	0.19
SF	-0.15	0.09	-0.42	0.001

Supplementary Table 12: Spearman correlation between mean lifetimes of latent brain states in the 2-back task and WM accuracy.

**Latent state**	**Session 1**		**Session 2**	
	r	*p*	*r*	*p*
SL	-0.2	0.03	-0.09	0.31
SH	0.35	0.001	0.26	0.004
ST	-0.2	0.03	-0.06	0.51
SF	-0.11	0.22	-0.42	0.001

Supplementary Table 13: Correlation between occupancy rates of latent brain states in the 2-back task and WM drift rates.

**Latent state**	**Session 1**		**Session 2**	
	r	*p*	*r*	*p*
SL	-0.24	0.009	-0.15	0.1
SH	0.35	0.001	0.28	0.002
ST	-0.22	0.01	-0.1	0.29
SF	-0.23	0.01	-0.34	0.001

Supplementary Table 14: Correlation between mean lifetimes of latent brain states in the 2-back task and WM drift rates.

**Latent state**	**Session 1**		**Session 2**	
	r	*p*	*r*	*p*
SL	-0.12	0.2	-0.14	0.12
SH	0.32	0.001	0.26	0.003
ST	-0.2	0.03	-0.04	0.63
SF	-0.18	0.05	-0.34	0.001

Supplementary Table 15: Correlation of posterior probabilities of latent brain states from training and test sessions (**Supplementary Results**: BSDS WM analysis using functional clusters of ICA components as ROIs).

		**Session 2 test state**				
		5	9	11	12	14
**Session 1 training state**	5	0.02	-0.27	-0.26	-0.42	**0.82**
	9	-0.39	**0.87**	-0.11	0.08	-0.29
	10	-0.20	0.01	**0.58**	-0.16	-0.10
	12	0.19	-0.23	0.37	-0.15	-0.15
	14	0.24	-0.28	-0.27	**0.56**	-0.39
		**Session 1 test state**				
		5	9	10	12	14
**Session 2 training state**	5	0.06	-0.39	-0.20	0.15	0.27
	9	-0.29	**0.87**	0.01	-0.24	-0.28
	11	-0.28	-0.12	**0.57**	0.39	-0.27
	12	-0.41	0.07	-0.16	-0.11	**0.56**
	14	**0.82**	-0.29	-0.11	-0.17	-0.37

**Matched latent state**	**Session 1**	**Session 2**
1	14	12
2	5	14
3	10	11
4	9	9
5	12	5

Supplementary Table 16: Correlation of posterior probabilities of latent brain states from training and test sessions (**Supplementary Results**: BSDS analysis with data regressed out 12 head motion parameters).

		Session 2 test state				
		1	4	7	11	13
**Session 1 training state**	1	-0.16	-0.18	-0.26	-0.42	**0.97**
	2	-0.45	-0.11	-0.05	**0.94**	-0.39
	3	-0.33	-0.09	**0.97**	-0.20	-0.22
	11	-0.19	**0.93**	-0.08	-0.18	-0.13
	13	**0.94**	-0.19	-0.37	-0.27	-0.31

		Session 1 test state				
		1	2	3	11	13
**Session 2 training state**	1	-0.16	-0.44	-0.33	-0.20	**0.94**
	4	-0.19	-0.11	-0.09	**0.93**	-0.19
	7	-0.26	-0.06	**0.97**	-0.09	-0.37
	11	-0.42	**0.94**	-0.19	-0.17	-0.25
	13	**0.97**	-0.38	-0.22	-0.14	-0.31

Matched latent state	Session 1	Session 2
1	2	11
2	1	13
3	11	4
4	3	7
5	13	1

Supplementary Table 17: Correlation between occupancy rates of latent brain states in the 2-back task and WM accuracy, using the temporal clustering approach (**Supplementary Results**: temporal clustering analysis).

Latent state	Session 1		Session 2	
	r	*p*	*r*	*p*
S1	0.02	0.87	0.04	0.65
S2	-0.02	0.87	-0.04	0.65

Supplementary Table 18: Correlation of posterior probabilities of latent brain states from training and test sessions (Supplementary Results: BSDS analysis on data from the HCP Relational Processing task).

		Session 2 test state			
		3	8	10	14
Session 1 training state	2	-0.24	**0.65**	-0.13	-0.27
	6	0.06	0.46	-0.1	-0.4
	8	-0.27	-0.24	**0.81**	-0.23
	12	**0.74**	-0.39	-0.19	-0.17
	15	-0.37	-0.35	-0.28	**0.93**

		Session 1 test state				
		2	6	8	12	15
Session 2 training state	3	-0.26	0.07	-0.26	**0.75**	-0.38
	8	**0.67**	0.42	-0.22	-0.4	-0.36
	10	-0.13	-0.09	**0.81**	-0.17	-0.29
	14	-0.28	-0.39	-0.22	-0.19	**0.94**

Matched latent state	Session 1	Session 2
1	2	8
2	8	10
3	12	3
4	15	14
	6	

Supplementary References

1. Rabiner, L. R. A tutorial on hidden Markov models and selected applications in speech recognition. *Proceedings of the IEEE* **77**, 257–286 (1989).

2. Bishop, C. M. *Pattern Recognition and Machine Learning* (Springer, 2006). `0-387-31073-8`.

3. Murphy, K. P. *Machine Learning: A Probabilistic Perspective* (The MIT Press, 2012).

4. Højen-Sørensen, P. & Hansen, L. K. Bayesian modelling of fMRI time series. In *Advances in Neural Information Processing Systems*, 754–760 (2000).

5. Eavani, H., Satterthwaite, T. D., Gur, R. E., Gur, R. C. & Davatzikos, C. *Unsupervised learning of functional network dynamics in resting state fMRI*, vol. 23, 426–37 (Springer Berlin Heidelberg, 2013).

6. Robinson, L. F., Atlas, L. Y. & Wager, T. D. Dynamic functional connectivity using state-based dynamic community structure: Method and application to opioid analgesia. *NeuroImage* **108**, 274–291 (2015).

7. Suk, H. I., Lee, S. W. & Shen, D. *A hybrid of deep network and hidden Markov model for MCI identification with resting-state fMRI*, vol. 9349, 573–580 (Springer International Publishing, 2015).

8. Everitt, B. S. *An Introduction to Latent Variable Models*, vol. 22 (Springer Netherlands, 1984).

9. Ghahramani, H. G. E., Z. The EM algorithm for mixtures of factor analyzers. Tech. Rep., Technical Rep ort CRG-TR-96-1, Department of Computer Science University of Toronto (1997).

10. Fox, E. B. *Bayesian nonparametric learning of complex dynamical phenomena*. Ph.D. thesis, Massachusetts Institute of Technology, Electrical Engineering and Computer Science, Massachusetts Institute of Technology, USA (2009).

11. Neal, R. M. *Bayesian learning for neural networks. Lecture notes in statistics* (Springer, 1996).

12. MacKay, D. J. C. *Bayesian Methods for Backpropagation Network* (Springer New York, New York, NY, 1996).

13. Bishop, C. M. Variational principal components. In *Proceedings Ninth International Conference on Artificial Neural Networks*, vol. 1, 509–514 (1999).

14. Ghahramani, Z. & Beal, M. J. Variational inference for Bayesian mixtures of factor analysers. In *Advances in Neural Information Processing Systems*, vol. 12, 449–455 (2000). `CIT:198801`.

15. Mackay, D. J. C. Ensemble learning for hidden Markov model. Tech. Rep., Cavendish Laboratory, University of Cambridge (1997).

16. Jordan, M. I., Ghahramani, Z., Jaakkola, T. S. & Saul, L. K. Introduction to variational methods for graphical models. *Machine Learning* **37**, 183–233 (1999).

17. Wainwright, M. J. & Jordan, M. I. Graphical models, exponential families, and variational inference. *Found. Trends Mach. Learn.* **1**, 1–305 (2008).

18. Beal, M. J. *Variational algorithms for approximate Bayesian inference*. Ph.D. thesis, Gatsby Computational Neuroscience Unit, University College London (2003).

19. Shirer, W. R., Ryali, S., Rykhlevskaia, E., Menon, V. & Greicius, M. D. Decoding subject-driven cognitive states with whole-brain connectivity patterns. *Cerebral Cortex* **22**, 158–165 (2012).

20. Fox, E., Sudderth, E. B., Jordan, M. I. & Willsky, A. S. Nonparametric Bayesian learning of switching linear dynamical systems. In Koller, D., Schuurmans, D., Bengio, Y. & Bottou, L. (eds.) *Advances in Neural Information Processing Systems 21*, 457–464 (Curran Associates, Inc., 2009).

21. Fox, E. B., Sudderth, E. B., Jordan, M. I. & Willsky, A. S. An HDP-HMM for systems with state persistence. In *Proceedings of the International Conference on Machine learning (ICML)* (2008).

22. Taghia, J. *et al.* Bayesian switching factor analysis for estimating time-varying functional connectivity in fMRI. *NeuroImage* **155**, 271–290 (2017).

23. Ryali, S. *et al.* Temporal dynamics and developmental maturation of salience, default and central-executive network interactions revealed by variational bayes hidden markov modeling. *PLOS Computational Biology* **12**, 1–29 (2016).

24. Vidaurre, D. *et al.* Spectrally resolved fast transient brain states in electrophysiological data. *NeuroImage* **126**, 81–95 (2016).

25. Allen, E. A. *et al.* Tracking whole-brain connectivity dynamics in the resting state. *Cerebral Cortex* **24**, 663–676 (2014).

26. Rashid, B. *et al.* Classification of schizophrenia and bipolar patients using static and dynamic resting-state fMRI brain connectivity. *NeuroImage* **134**, 645–657 (2016).

27. Cai, W., Chen, T., Szegletes, L., Supekar, K. & Menon, V. Aberrant time-varying cross-network interactions in children with attention-deficit/hyperactivity disorder and the relationto attention deficits. *Biological Psychiatry: Cognitive Neuroscience and Neuroimaging* **3**, 263–273 (2018).

28. Bellec, P., Rosa-Neto, P., Lyttelton, O. C., Benali, H. & Evans, A. C. Multi-level bootstrap analysis of stable clusters in resting-state fMRI. *NeuroImage* **51**, 1126–1139 (2010).

29. Smith, R., Keramatian, K. & Christoff, K. Localizing the rostrolateral prefrontal cortex at the individual level. *NeuroImage* **36**, 1387–1396 (2007).
